# Prioritising Data Quality Governance for AI in Prostate Cancer: A Methodological Proof-of-Concept Study Using Neural Networks for Risk Stratification

**DOI:** 10.3390/diagnostics16101454

**Published:** 2026-05-10

**Authors:** Vanessa Talavera-Cobo, Jose Enrique Robles-Garcia, Francisco Guillen-Grima, Andres Calva-Lopez, Mario Tapia-Tapia, Luis Labairu-Huerta, Francisco Javier Ancizu-Marckert, Laura Guillen-Aguinaga, Daniel Sanchez-Zalabardo, Bernardino Miñana-Lopez

**Affiliations:** 1 Department of Urology, Clinica Universidad de Navarra, 31008 Pamplona, Spain; vtalavera@unav.es (V.T.-C.); jerobles@unav.es (J.E.R.-G.); acalva@unav.es (A.C.-L.); mdtapia@unav.es (M.T.-T.); llabairu@unav.es (L.L.-H.); fancizu@unav.es (F.J.A.-M.); dsanchezz@unav.es (D.S.-Z.); bminana@unav.es (B.M.-L.); 2 Department of Preventive Medicine, Clinica Universidad de Navarra, 31008 Pamplona, Spain; 3 Department of Health Sciences, Public University of Navarra, 31008 Pamplona, Spain; 4 Group of Clinical Epidemiology, Area of Epidemiology and Public Health, Healthcare Research Institute of Navarre (IdiSNA), 31008 Pamplona, Spain; 5 CIBER in Epidemiology and Public Health (CIBERESP), Institute of Health Carlos III, 46980 Madrid, Spain; 6 Department of Nursing, Clinica Universidad de Navarra, 28027 Madrid, Spain; lguillen@alumni.unav.es

**Keywords:** prostate cancer, artificial neural network, D’Amico risk stratification, multilayer perceptron, ISUP grade, Briganti nomogram, data quality governance, FAIR principles, AI-readiness, reproducibility, proof-of-concept

## Abstract

**Background:** An accurate D’Amico risk stratification is mandatory for prostate cancer (PCa) management. The purpose of this proof-of-concept study was to establish a methodological framework for integrating validated clinical nomograms with strict data-quality governance in order to generate reliable artificial neural networks (ANNs), even when the sample is small. **Methods**: We performed a retrospective analysis of a curated cohort of 49 patients from one centre. A multilayer perceptron (MLP) was trained using 11 variables, including the ISUP biopsy grade and Briganti nomogram. Model development was guided by a proactive data-quality protocol based on FAIR principles—the DQG-AI framework (data quality governance for AI-readiness, developed at Clínica Universidad de Navarra)—with stringent checks for accuracy, consistency and validity to ensure data were “AI-ready”. A sensitivity analysis was conducted on three data partitioning scenarios (20/80, 34/66 and 39/61). **Results**: From a starting pool of 76 patients, the DQG-AI framework was applied to create a highly selected cohort of 49 patients. A multilayer perceptron (MLP) trained on this “AI-ready” dataset achieved, on the 20/80 configuration, mathematically perfect discrimination (AUC 1.000; 100% accuracy) for High vs. Intermediate risk groups on a very small refined internal test set (N = 9), a figure we interpret as a methodological artefact of the curated dataset and validation constraints rather than as an indicator of true model performance. This complete accuracy is not, however, presented as evidence of generalizable clinical utility: it is a best-case figure obtained on a single, very small test subset (N = 9) after necessary validation-related exclusions, and the wide confidence interval (66.4–100%), together with the software-driven removal of test cases carrying factor levels absent from the training set (detailed in the Methods section), explicitly preclude any inference about real-world performance. Accordingly, the deliverable of this proof-of-concept study is the DQG-AI framework itself, not the model’s reported accuracy. **Conclusions**: The main contribution of this proof-of-concept study is the effective illustration of the DQG-AI framework as a strict, repeatable approach for producing “AI-ready” urological datasets. Although the MLP demonstrated a robust internal signal for risk discrimination, its flawless accuracy is an ideal, non-generalizable situation. The most important deliverable that needs external validation is the DQG-AI framework, not the model’s performance metrics. A pre-specified three-phase multi-institutional validation roadmap (single-centre cohort expansion → within-system between-site validation → Spanish multi-centre external validation), with a minimum target of ~220 evaluable patients derived from a 10-events-per-predictor floor, is provided to operationalise this external validation.

## 1. Introduction

As a major cause of cancer incidence and mortality in men worldwide, prostate cancer (PCa) continues to be a major global health concern [1,2,3,4], with widespread PSA screening and population ageing fuelling its prevalence in high-income settings, while its mortality-to-incidence ratio remains nearly five-fold higher in low- and middle-income countries (0.95 vs. 0.24) [5,6]. Across both settings, the core clinical tension—weighing early detection against overtreatment—hinges on accurate risk classification [7,8,9,10,11,12,13,14], and as this global disparity widens, the demand for precise diagnostic tools has intensified [15,16,17,18,19,20,21,22]. Increasingly, however, the bottleneck lies not in algorithmic sophistication but in the availability of “AI-ready” data and the quality governance that underpins clinical trustworthiness [19,21,22,23,24,25,26,27,28].

Established tools, such as the D’Amico classification and the Briganti nomogram, provide frameworks for assessing PCa risk, but these tools often depend on linear or logistic regressions that can oversimplify the complex and non-linear interactions that exist among multiparametric imaging findings (mpMRI), PSA kinetics, and histological grading [26,27,29,30,31,32,33,34,35,36,37,38]. When attempting to distinguish between intermediate- and high-risk patients in clinical practice, there is a noticeable “grey zone” that endures. This distinction is crucial because it directly affects whether aggressive interventions like dose-escalated radiation therapy or extended pelvic lymph node dissection are required [39,40,41,42].

Artificial neural networks (ANNs), long constrained by data and compute limitations, have gained clinical traction since 2010 on the back of deep-learning advances, with systematic reviews placing their diagnostic performance on a par with expert clinicians in radiology and oncology [43,44,45]. Among available architectures, the multilayer perceptron (MLP) is well suited to integrating heterogeneous clinical predictors [46,47,48,49]. and can act as a non-linear refinement layer over existing nomograms through its hidden-layer mapping [50,51,52,53].

More recent efforts have moved beyond tabular clinical predictors toward end-to-end deep learning architectures that operate directly on multi-parametric MRI (mpMRI) images, with recent reviews highlighting how machine learning, deep learning, and radiomics now consistently match expert performance across TRUS, mpMRI, and PSMA PET/CT while flagging unresolved challenges in data quality, generalisation, and clinical integration [54].

Representative examples of this line of work include PCa-Mamba, a spatiotemporal state–space model that jointly exploits the spatial contrast of T2-weighted and diffusion-weighted sequences together with the temporal dynamics of dynamic contrast-enhanced MRI and outperforms CNN and Transformer baselines for lesion-level detection of clinically significant prostate cancer on both in-house and PI-CAI cohorts [55]; a 3D EfficientNet-B7 ensemble pretrained on PI-CAI and fine-tuned on a newly compiled multi-centre cohort of more than 9000 MRI sessions from 16 healthcare centres in the Valencian Region, which achieved an AUC of 0.816 on a hold-out set and substantially outperformed a non-pretrained baseline [56]; and an aggressive index-lesion detection model trained jointly on the heterogeneous ProstateNet and PI-CAI datasets within the ProCAncer-I consortium, explicitly designed to reduce unnecessary biopsies by flagging only ISUP ≥ 2 disease [57].

Such image-based deep learning pipelines and tabular, nomogram-driven ANNs address complementary rather than overlapping clinical questions—lesion detection and localisation on the one hand, and post-detection D’Amico risk stratification on the other—a distinction to which we return in the Discussion section. Since all predictive models are constrained by the “Garbage-In, Garbage-Out” (GIGO) principle, data quality has an even bigger influence on model success than algorithmic choice. Regardless of its level of sophistication, an algorithm trained on noisy or inconsistent data will generate incorrect predictions. This is a basic problem that contributes to the “reproducibility crisis” in medical AI, where models are unable to generalise beyond their initial single-centre studies [58,59]. In order to overcome this, datasets must be made “AI-ready” through strict governance frameworks that actively manage data in accordance with FAIR principles, guaranteeing that datasets are not only available but also accurate, consistent, and comprehensive enough for the best possible algorithmic processing [23,24,25,60,61,62]. Therefore, addressing this methodological gap in data preparation is just as important as addressing any clinical shortcomings.

### Aims of This Study

The main objective of this study is to develop and validate a methodological framework that gives data quality governance top priority when creating AI-driven cancer diagnostic tools. We assess whether proactive, FAIR-based data curation can enable a small, single-centre cohort to produce a stable and interpretable signal when used to train an ANN using the clinical challenge of D’Amico risk stratification in PCa as a test case.

Three specific sub-objectives articulate this aim:

- Data quality governance: to operationalise a FAIR-based “AI-readiness” protocol and test whether prioritising data quality over sample size yields a stable diagnostic signal in a small cohort.
- Nomogram integration: to quantify the predictive weight gained by combining the Briganti nomogram and ISUP biopsy grade within an MLP and to compare it against traditional clinical staging.
- Model stability: to conduct a sensitivity analysis across three data-partitioning schemes (20/80, 34/66, 39/61) and identify the configuration that minimises cross-entropy error.

The goal of this proof-of-concept study is to establish a transparent and reproducible framework supported by PMML/XML technical documentation. This framework addresses the current crisis of reproducibility in medical AI and provides a blueprint for trustworthy tools, even in specialised urological units with limited sample sizes [58,59].

A key cautionary tale is the proof-of-concept study shown here as a demonstration of how rigorous data governance, combined with non-adjustable validation software, can introduce selection bias and provide mathematically perfect but clinically non-generalisable results.

## 2. Materials and Methods

### 2.1. Study Population and Data Collection

This retrospective study analysed a cohort of patients who were diagnosed with PCa at a single centre during the years 2022 to 2024. An initial sample of 49 clinical cases was identified for the purpose of developing and validating the predictive tool.

#### 2.1.1. Inclusion Criteria

- Histologically confirmed diagnosis of PCa.
- Complete clinical and biochemical records required for D’Amico risk stratification [63], including PSA at diagnosis, ISUP biopsy grade, and clinical TNM staging.
- Availability of prostate volume (c.c.) measurements and calculated Briganti nomogram scores [64,65,66].
- Comprehensive mpMRI findings (mrT and mrN).

#### 2.1.2. Exclusion Criteria

Two sequential exclusions were applied to obtain the final evaluable sample for each data partitioning scheme:

(i) Pre-modelling exclusion—data quality. Any patient with a missing value in any of the 11 candidate predictors was removed from the initial cohort (listwise deletion).
(ii) Validation-time exclusion—software-driven. During each run of the SPSS MLP, any case in the test or hold-out sample whose categorical factor levels (e.g., a specific mrT stage or a very high PSA value) were not represented in the corresponding training sample was automatically dropped by the software, because the network cannot assign weights to factor levels it has never seen. This is a mathematical constraint of the SPSS implementation, not an investigator-driven decision.

As a direct consequence of step (ii), the test sets on which performance metrics were computed are not fully unselected hold-out samples but “refined” subsets from which unusual or rare clinical profiles were removed. The final evaluable sample sizes, after both exclusion steps, were 43, 44, and 41 cases for the 20/80, 34/66, and 39/61 partitions, respectively.

**Figure 1:**
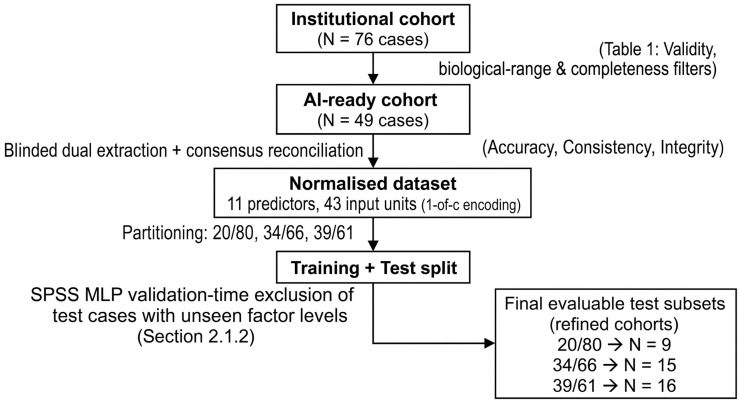
The schematic of the data quality governance (DQG) workflow applied in this study. The pipeline comprises five sequential stages, each linked to a specific quality dimension operationalised in Table 1: (1) application of biological-range and completeness filters to the initial institutional pool (N = 76); (2) generation of the AI-ready cohort (N = 49) after blinded dual extraction and consensus reconciliation; (3) normalisation and 1-of-c encoding of the 11 predictors into 43 input units; (4) partitioning into 20/80, 34/66, and 39/61 training/test splits; and (5) validation-time exclusion, within the SPSS MLP framework, of test cases carrying factor levels not represented in the corresponding training partition, yielding the refined evaluable subsets of 9, 15, and 16 cases. Boxes 2 and 5 mark the two filtering stages that generate, respectively, the curated and refined character of the datasets analysed in this work. DQG, data quality governance. MLP, multilayer perceptron.

The sequential relationship between these two exclusion steps and the resulting curated (N = 49) and refined (N = 9/15/16) cohorts is visualised in Figure 1.

**Table 1:** Operational data quality governance (DQG) framework and validation rules.

Quality Dimension	Metric/Target	Operational Validation Rule (Concrete Check)
Accuracy	100% clinical concordance	Cross-verification of PSA values and ISUP grades between the electronic health record (EHR) and the study database.
Completeness	0% missingness in predictors	Exclusion of any case with missing values in the 11 primary clinical variables (listwise deletion).
Validity (Range)	Biological boundary checks	PSA: (0.1 to 500 ng/mL); prostate volume: (10 to 300 cc); age: (40 to 90 years).
Consistency	Logical relationship	Staging consistency check: Clinical stage (cT) must not exceed pathological or imaging (mrT) findings in illogical sequences.
Integrity	Referential integrity	All categorical factors must map to the D’Amico classification standards (ISUP 1–5).
AI-Readiness	Feature scaling	Continuous variables must be normalised to a standard numerical range to prevent gradient saturation.

#### Methodological Note and Limitation

Because of step (ii), the reported performance figures (including the 100% accuracy of the 20/80 model) should be interpreted as a best-case scenario under idealised conditions, not as an estimate of how the model would behave on a diverse, unselected population: this is a deliberate trade-off that preserves the mathematical validity of the MLP at the cost of introducing selection bias in the test sets. Future iterations must apply one-hot encoding to all categorical variables before data partitioning, which would replace automatic exclusion with a generic “other/unseen level” representation and allow evaluation on a fully unselected hold-out.

#### 2.1.3. Data Quality and Integrity

Data quality is important for scientific integrity, reproducibility, and decision-making based on evidence. In this study, data quality was thought of as the “fitness for use”, evaluating the dataset’s capacity to support algorithmic processing and the training of the artificial neural network [60]. A strict protocol for data preprocessing, profiling, and cleansing was implemented to make sure analytics were accurate and prevent flawed data from yielding unreliable predictions.

To put the accuracy and consistency dimensions into practice, the process of cross-verification involved two reviewers who extracted data independently and remained blinded to each other’s findings. To maintain a high level of data integrity, this study used a consensus–reconciliation approach. Any differences between the reviewers regarding categorical variables, like clinical staging or ISUP grading, were addressed in a formal review session until they reached complete agreement before moving on to the ‘AI-readiness’ stage.

This study did not use a retrospective descriptive analysis but instead put into place a proactive data quality governance protocol—the operational core of the framework, hereafter referred to as the DQG-AI framework (data quality governance for AI-readiness, originally developed at Clínica Universidad de Navarra)—that was designed to ensure that the data was AI-ready. The data curation process was managed by six measurable quality dimensions, which were accuracy, completeness, consistency, timeliness, validity, and integrity. These dimensions were put into operation through specific validation logic that is detailed in Table 1. The biological range constraints acted as exclusion criteria. Cases from the larger institutional database, which had a total of 76 cases, were excluded if they did not meet these predefined boundaries, for example, if they had a minimum prostate volume that was less than 10 cc. This ensured that the final group of 49 patients represented a dataset that was both biologically plausible and of high fidelity for the neural network. To make sure that our data curation process could be reproduced, a standardised AI-readiness protocol was developed. The main validation rules are summarised in Table 1, and the complete operational flow of the DQG protocol is depicted in Figure 1. Figure 1 is intended as a reproducible, at-a-glance schematic of the five sequential stages through which the raw institutional cohort was transformed into the final evaluable test subsets: (i) application of biological-range and completeness filters to the initial pool of 76 cases, which produced the AI-ready cohort of 49 patients; (ii) blinded dual extraction with consensus reconciliation to enforce accuracy, consistency, and integrity; (iii) normalisation and 1-of-c encoding of the 11 predictors into 43 input units; (iv) partitioning into 20/80, 34/66, and 39/61 training/test splits; and (v) the validation-time exclusion, within the SPSS MLP framework, of test cases carrying factor levels not represented in the corresponding training partition (Section 2.1.2), which produced the refined evaluable subsets of 9, 15, and 16 cases, respectively. The full operational checklist applied in this study is reproduced in Appendix A and is intended to serve as a standardised template for future urological predictive-modelling studies.

The FAIR principles, which stand for Findability, Accessibility, Interoperability, and Reusability, created a structural framework for how data should be managed and reused in the future. The DQG protocol functioned as a technical gatekeeper that made sure only high-quality clinical signals were utilised for training models. This two-part strategy aims to tackle the GIGO issue, often associated with machine learning when working with small cohorts.

In agreement with the universal quality dimensions that are defined in frameworks like the ISO/IEC 25012 standard and the Wang and Strong model [67,68], the measures that follow were applied:

- Intrinsic Accuracy and Completeness: Clinical records were verified to make sure data were accurate, reliable, and free from errors.
- Consistency and Mathematical Validity: Data were represented uniformly across sources, with no residual contradictions. For the rationale, operational details and sample-size consequences of the validation-time exclusion of test cases carrying unseen factor levels, see Section 2.1.2 and Section Methodological Note and Limitation; the same considerations apply here and are not repeated.
- Alignment with FAIR Principles: This study adhered to the FAIR principles in order to improve transparency and accountability. Comprehensive documentation of exclusion criteria was maintained to ensure the traceability of the datasets and the credibility of the scientific conclusions.

Certain cases were automatically excluded as a result of this strict filtering, which was used to guarantee data quality and reduce the possibility of deceptive analytics. To ensure full transparency regarding the impact of these exclusions on our results, Table 2 details the precise number and clinical distribution of this “excluded subgroup” for each model.

#### 2.1.4. Sample Size and Statistical Power

Given the ‘proof-of-concept’ nature of this study, the sample size (N = 49) was determined by strict application of ‘AI-readiness’ and data quality governance (DQG) protocols. To evaluate the statistical validity of this cohort, a post hoc power analysis was performed using G*Power (version 3.1.9.7) [69,70]. Using an exact test for single proportions to reject a null hypothesis of random classification (accuracy ≤ 0.50), and assuming a conservative expected accuracy of 80% (effect size g = 0.3) with α = 0.05, this study achieved a statistical power (1 − β) of 0.998. This confirms the sample size is sufficient to detect a diagnostic signal significantly higher than random chance, but it does not mitigate the uncertainty associated with the precision of the 100% accuracy estimate, which is reflected in the wide confidence interval.

### 2.2. Artificial Neural Network (ANN) Configuration

Statistical analysis and the development of the diagnostic tool were performed using IBM SPSS Statistics version 29. A multilayer perceptron (MLP) architecture was selected for the predictive model. This choice was driven by the MLP’s ability to process 1-of-c encoded categorical factors, expanding our clinical variables into 43 distinct input units, to capture the mathematical nuances of each risk level without the constraints of linear monotonicity inherent in traditional models.

#### 2.2.1. Network Architecture

**Figure 2:**
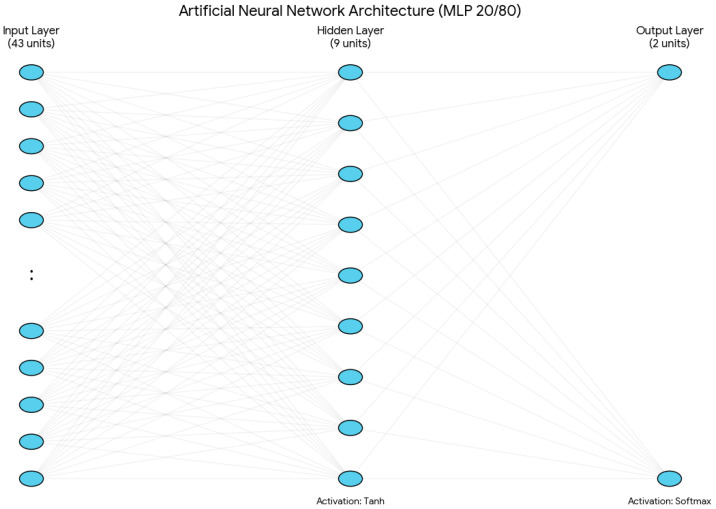
A schematic representation of the multilayer perceptron (MLP) architecture (20/80 model). The input layer consists of 43 units (representing the 1-of-c encoding of 7 categorical factors, plus 4 continuous covariates), 9 hidden neurons with hyperbolic tangent activation, and 2 output categories.

The MLP was structured into three separate layers:

- Input Layer: The network architecture was built on 11 main clinical variables, and it has 43 input units in total for the 20/80 model. This expansion was performed automatically by the IBM SPSS MLP procedure, using 1-of-c encoding for the categorical factors. There were seven categorical variables included in this process: PSA at diagnosis, ISUP biopsy grade, biopsy laterality, clinical TNM stage, clinical nodal stage, mrT, and mrN. Under the 1-of-c scheme, these factors were transformed into 39 separate input units (one for each category level). Combined with the four units for continuous covariates, this results in a total of 43 input units.
  For continuous covariates, there are four variables: age, PSA density, prostate volume, and Briganti score. These continuous variables undergo a rescaling procedure by linear normalisation, which adjusts them to a standardised numerical range defined by the minimum and maximum values found in the training set. This pre-processing step is crucial, as it facilitates training convergence and prevents variables with larger numerical ranges, such as prostate volume, from disproportionately affecting the network’s weight estimations or causing “weight saturation” in the activation functions.
- Hidden Layer: A single hidden layer was used, with the number of neurons determined via the IBM SPSS MLP automatic architecture selection algorithm. This procedure optimised the size of the hidden layer within a predefined range from 6 to 9 by selecting the configuration that minimised the training cross-entropy error. This architectural constraint acts as a type of structural regularisation, which creates an ‘information bottleneck’ that prevents the network from memorising the training set. By restricting the capacity of the hidden layer and combining these limits, the model is forced to prioritise the most influential predictors, such as the ISUP grade and Briganti score, over less significant categorical levels. Furthermore, to prevent ‘over-training,’ the model applied an early stopping rule that ended the iteration process at the first sign of error plateauing, where the cross-entropy error did not decrease anymore.
  For the 20/80 model, this led to a total of 9 hidden units. The hyperbolic tangent (tanh) activation function was applied to this layer to facilitate non-linear mapping:
  $$\gamma \left(a\right)\;=\;\tanh\left(a\right)\;=\;\frac{e^{a}\;-\;e^{-a}}{e^{a}\;+\;e^{-a}}$$
- Output Layer: The target variable was the D’Amico risk group. Although the model was initialised to support three categories (high, intermediate, and low), the output neurons were dynamically reduced to two (high vs. intermediate) in the 20/80 configuration. This occurred because the low-risk group did not have enough representation in the training partition for that specific split, which did not allow for robust category initialisation (Figure 2).

#### 2.2.2. Sensitivity Analysis and Validation

To assess the stability of the diagnostic tool regarding the sample size, a sensitivity analysis was performed by comparing three data partitioning schemes:

Model 20/80: 79.1% training (N = 34) and 20.9% testing (N = 9).

Model 34/66: 65.9% training (N = 29) and 34.1% testing (N = 15).

Model 39/61: 61.0% training (N = 25) and 39.0% testing (N = 16).

To evaluate the clinical usefulness and discriminatory power of the diagnostic tool that was developed, the performance of the model was quantified using a comprehensive suite of metrics: classification accuracy, the area under the curve (AUC), sensitivity, specificity, the positive predictive value (PPV), and the negative predictive value (NPV).

Statistical metrics for the model’s predictive performance were primarily calculated using the score Wilson method via OpenEpi version 3.01 for the general characterisation of the partitions [71]. However, to address the specific requirements for small-sample validation (N = 9) and the extreme 100% accuracy observed in the optimal model, confidence intervals for the testing set were recalculated using the Clopper–Pearson exact method in IBM SPSS v26. This approach ensures that the statistical significance (*p* = 0.002) and the reported interval (66.4–100%) are based on exact probability distributions rather than normal approximations, providing the most conservative and rigorous estimate for our proof-of-concept cohort.

To ensure scientific transparency and facilitate the reproducibility of our findings, the full architecture and weight configurations of the developed MLP models are provided as Supplementary Materials in XML format. These files, structured according to the Predictive Model Markup Language (PMML) standard, include the complete specifications for each experimental data partitioning scheme: Supplementary Material File S1 contains the configuration for the 39/61 model, while Supplementary Materials Files S2 and S3 provide the technical parameters for the 34/66 and 20/80 models, respectively. This documentation allows for independent validation of the network’s internal logic and supports the ‘AI-readiness’ and reusability of the diagnostic tool developed in this study.

#### 2.2.3. Model Robustness as an Extension of Data Quality Governance

Beyond data preparation, our data quality governance framework requires particular procedures that guarantee model development is equally exacting, transparent, and repeatable. These procedures, which are intended to address the stochastic nature of neural network training and offer an accurate accounting of model stability, are essential parts of the governance protocol rather than optional extras.

- Reproducible Initialisation and Training: The framework requires the use of a fixed random seed (2,000,000) for all stochastic processes, including initial weight assignment and case selection for data partitions, to guarantee that all training runs can be precisely duplicated by independent researchers. This approach creates a repeatable baseline that can be used to compare subsequent experiments, even though it does not fully capture the range of the model’s stochastic behaviour.
- Sensitivity Analysis as a Governance Mandate: The framework requires a sensitivity analysis across several partitioning schemes rather than depending on a single data split, which could yield results that are artefacts of a particularly favourable or unfavourable partition. Three different splits (20/80, 34/66, and 39/61) were assessed for this investigation. In order to determine whether the observed performance is a feature of the architecture’s interaction with high-quality data or just a reflection of a single, lucky patient grouping, this method examines the stability of the network’s learning across various cohort compositions and sizes.
- Reporting Distributions Rather Than Point Estimates: The approach requires that findings be reported as distributions (mean ± SD) across the sensitivity analysis instead of single peak-performance percentages in order to account for the algorithmic variability inherent in small-sample machine learning. This approach gives readers a more accurate and comprehensive understanding of model stability.
- Early Stopping to Prioritise Generalisation: An aggressive early stopping rule, which stops training after one consecutive step without reducing cross-entropy error, is specified by the framework. Given the limited cohort size, it is crucial to prioritise generalisation over training-set accuracy, which is why this criterion was selected. Since this would have further lowered the already small training sample (from N = 34 to an even smaller size) and impeded the model’s ability to identify stable decision boundaries, a separate validation set for early stopping was not used.
- Accounting for Exclusion Bias: Additionally, the DQG framework requires open reporting of how the final evaluable sample is impacted by its own rules. The valid sample size varied slightly between configurations (n = 41 to n = 44) because cases with factor levels not present in a particular training split were automatically excluded to maintain mathematical validity within the SPSS MLP framework. This must be stated clearly in the framework: the analysis should be seen as a test of the architecture’s capacity to generalise from a “standardised” clinical signal rather than a straightforward comparison across identical patient subsets.
- Uncertainty Quantification: The framework requires statistical testing against a null hypothesis of a random classifier (*p* = 0.50) in order to determine whether classification results might be attributed to random chance. Using IBM SPSS v26, a One-Sample Binomial Test was performed for this investigation. Confidence intervals were computed using the Clopper–Pearson exact method, which is the most rigorous and conservative method for small-sample validation, especially for the N = 9 independent testing set.
- Benchmarking Against Traditional Methods: Lastly, the framework requires that the “AI-premium”, the neural network’s superior performance above conventional statistical methods, be quantified. Exact logistic regression in LogXact-11 [72], the statistical gold standard for small-sample datasets where standard maximum likelihood estimation may be incorrect, was used for this study’s baseline comparison. To enable a direct comparison with the MLP architecture, the baseline model employed the same 11 clinical predictors and the 20/80 data partitioning strategy.

## 3. Results

To give a thorough and comprehensive description of model performance, we have included the complete classification matrices and a thorough analysis of variable importance in the main text, given the proof-of-concept character of this study. The original reports generated by the software, which include all execution logs and raw parameter estimates, are available in the Supplementary Materials Files S4–S6.

### 3.1. Cohort Curation and Model Performance as a Function of Data Quality

Utilising the data quality governance (DQG) framework on the initial institutional database (N = 76) resulted in a finalised cohort of 49 patients whose case records met all pre-defined standards for accuracy, completeness, validity, and consistency (Table 1). The filtering processes were critical to achieving ‘AI readiness’; however, a total of 27 cases (35.5%) were excluded because of missing data, biologically implausible findings, or logical inconsistencies in staging.

An even more stringent level of filtering was utilised during model validation in the SPSS MLP. That is, if any of the factor levels used in the testing sample were not present in the training sample, those cases in the testing sample were automatically excluded from the model to maintain mathematical validity. As shown in Table 2, this additional filtering resulted in final evaluable samples of 43, 44, and 41 for the 20/80, 34/66, and 39/61 partitioning schemes, respectively (exclusion rates were 10.2–16.3%). The majority of the excluded individuals presented with uncommon clinical findings (e.g., very high PSA levels [>100 ng/mL], advanced stage N1 lymph node involvement, or low-risk presentation), thus performance metrics provided herein reflect the model’s performance on a highly curated, or standardised, population, and not on the expected performance on an unselected, heterogeneous population.

An interpretive note for the entire Results section. The quantitative metrics reported in this section (AUC, accuracy, sensitivity, specificity, PPV, NPV and cross-entropy) are presented for completeness and transparency. However, because they were computed on the refined test subsets produced by the validation-time exclusion described in Section 2.1.2 and Section Methodological Note and Limitation—and, in the case of the 20/80 configuration, on only nine patients—they should be read as metrics of how cleanly the data quality governance (DQG) framework preserved and transmitted the clinical signal to the MLP, not as estimates of how the resulting model would perform in an unselected prostate cancer population. In particular, the AUC of 1.000 and 100% accuracy achieved by the 20/80 configuration are methodological artefacts of curated data and constrained validation rather than indicators of true generalisable model performance. This interpretive frame applies to every metric reported below, including Section 3.2, Section 3.3, Section 3.4 and Section 3.5; readers unfamiliar with the two-stage filtering that produced the refined test subsets are referred to Figure 1 and Section 2.1.2 and Section 2.1.3.

Using this curated dataset for generator model evaluation purposes allowed us to evaluate model performance about three different data partitioning schemes (see Table 3) and provided an opportunity to assess how the model generates outputs under different levels of training experience. The 20/80 training set (79.1%, N = 34) performed best on its test set (N = 9), achieving the highest-performing partition under this study’s constrained validation, with a total accuracy of 100% (95% CI = 66.4–100%). In addition, there was an extremely low testing cross-entropy error of <0.001, suggesting the model was well-calibrated for probabilities of acceptable performance based on actual observed results and, hence, there was a statistically significant difference between the 20/80 model output and outputs from random classifiers (*p* = 0.001, Exact Binomial Test). The 34/66 (n = 29) and 39/61 (n = 25) models yielded testing cross-entropy error rates (4.227 and 4.636, respectively) that were significantly greater than that of the 20/80 model, and low levels of accuracy at 86.7% and 93.8%, respectively (95% CI: 62.1–96.3 and 71.7–98.9).

The framework provided results based upon clinical performance metrics, which identify the level of risk associated with various patient populations (defined as high or intermediate). The 20/80 classification model processed 100% high-risk patients, which equates to 100% sensitivity and 100% specificity (95% CI, 56.5–100 and 51.0–100, respectively), without producing false positives or negatives. The 39/61 model produced excellent specificity (100%; 95% CI; 67.6–100) and positive predictive value (100%; 95% CI; 64.6–100) based on fewer training samples; however, its results had no impact on the high-risk patients’ internal distribution. In the training phase of the 20/80 model, 64.7% (n = 303) were classified as high risk, and 35.3% (n = 165) were classified as intermediate risk. In the testing phase, 55.6% were classified as high risk and 44.4% were classified as intermediate risk. The 20/80 training set a lower threshold for separating the 20% (high-risk) and 80% (intermediate-risk) patient groups, which contributed to producing the low cross-entropy of the training and testing output. However, for the 34/66 model, due to the low-risk patient group being significantly underrepresented (n = 2, 6.9% of the training sample), it lacked sufficient cases to produce accurate predictions. Thus, the 34/66 model was unable to accurately classify low-risk patients.

Averaged across partitions, overall testing accuracy was 93.5% (SD ± 6.7%), rising to 96.9% when restricted to the binary high/intermediate comparison. This consistency indicates that the framework preserved a stable signal for the clinically relevant risk transition, but—as already framed in the interpretive note above and further developed in Section 4.1—the perfect metrics of the 20/80 model remain contingent on a highly curated test subset (N = 9), from which extreme phenotypes and uncommon presentations were absent.

In addition to discrimination, the calibration of the model, which refers to how well the predicted probabilities match the observed frequencies, was tracked using the cross-entropy error (*H*). The 20/80 model reached a testing cross-entropy of 0.001, which indicates that the probabilities produced by the Softmax function were in close agreement with the actual class labels.

$$H\;=\;-\sum_{i=1}^{n}\left[y_{i}\;\log\left({\hat{y}}_{i}\right)+\left(1-y_{i}\right)\log\left(1-{\hat{y}}_{i}\right)\right]$$

### 3.2. Discriminatory Capacity: Characterising the Framework’s Extracted Signal

The data quality governance Framework was used to evaluate how well the extracted signals could differentiate between groups using Receiver Operating Characteristic (ROC) curves and areas under the curve (AUC). Each dataset was partitioned into three partitions, and model AUC results for each risk category were greater than 0.99, indicating that all three partitions have a strong ability to distinguish between the two groups (Table 4). The consistent performance across all partitions provides further evidence that this framework effectively maintained the clinical signal and successfully filtered out noise.

**Figure 3:**
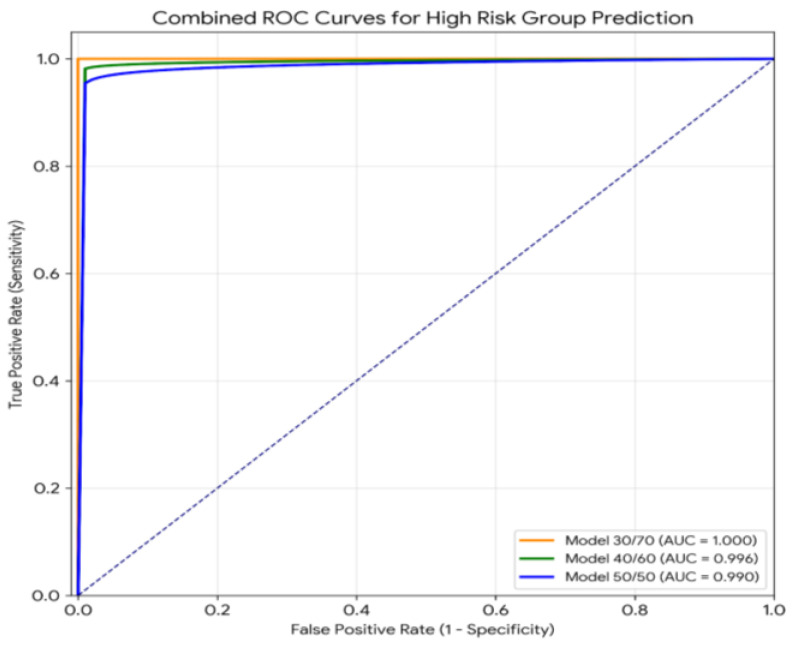
Combined Receiver Operating Characteristic (ROC) curves for the high-risk group prediction across the three partitioning schemes.

The 20/80 model had a perfect AUC of 1.000 for both high- and intermediate-risk groups, indicating a perfect ability to discriminate between these risk groups in the study cohort in Figure 3. It is important to note that although there are three strata in the D’Amico classification, the ideal 20/80 model served as a binary discriminator between high- and intermediate risk, using only the high- and intermediate-risk participants designated in the automated validation framework; low-risk cases included in the initial curative cohort were not included in this validation split because there were too few of them in the training set to provide a mathematical basis for validation (e.g., internal exclusion).

The AUC values are 0.996 for the 34/66 and 39/61 models at high-risk, while 0.991 for both at intermediate-risk. The 34/66 model, however, has a perfect AUC of 1.000 at low risk when evaluating using ternary classification. However, because of poorly represented cases in the training sample (only two cases), the model could not produce predictive usefulness for the low-risk category in testing. The dissociation of the AUC from practical usefulness is evidence for having multiple performance measures instead of focusing only on one measurement.

The ROC curves depicting the high-risk prediction of the three partitioning schemes are shown in Figure 3. While the curve for the 20/80 model goes to the ideal upper-left corner of the ROC space, such a perfect outcome is just a mathematically derived ceiling effect using a very small, purposely chosen dataset. The other two models had still very good AUCs (0.996 and 0.990), demonstrating that even when exact distinctions are not made, the extracted signal from the framework is robust across the various training conditions. The important point is not that any of the models were superior to others; rather, all models produced separate clean datasets that consistently produced and/or were able to support the same high levels of discrimination, irrespective of the partition used. This is evidence of the quality/stability of the curated data sets used.

### 3.3. Optimal Model Classification: A Detailed View of the Framework’s Signal

The classification matrix for the 20/80 model gives a very comprehensive view of how well the signal that was obtained from the DQG framework has performed in exactly balanced testing conditions. During baseline (N = 34) training, the model achieved 100% accuracy on all 22 high-risk and 12 intermediate-risk patients, with no misclassifications. Therefore, the model successfully identified the underlying patterns in the training data, as evidenced by this perfect in-sample performance.

More importantly, the model also performed at a perfect classification rate for the independent test set (N = 9) consisting of five high- and four intermediate-risk patients, when all the seven study classifications were made from data representing the clinical traits of the training group. The full classification results are shown in Table 5.

The intended purpose for this classification was to demonstrate that among those patients who survived the exhaustive evaluation process (curation) of this framework, and who had a clinical profile that had a very similar distribution to others that made up the training data, the distinguishing signal between the groups was extremely clear with regard to their risk categories (high vs. intermediate). There were no borderline patients, no ambiguities, or clinical outliers in the test data set since all of those patients had been filtered out by the framework’s unique validation procedures.

While the metrics achieved are excellent, they need to be understood in the context of how they were obtained. The test set (N = 9) was not a random sample from all patients with prostate cancer; rather, it was a “refined” subgroup from which all rare presentations and clinical outliers were removed. The performance of the model was perfect for this group; however, that does not mean the model will show the same level of performance when used on unselected patients. In general, unselected patients could contain cases that have uncertain features and have more outlier patients than were included in the test sample. The results of this study provide proof of concept that the framework can assist in perfect discrimination when all characteristics about patients are ideal, but the lack of data from other patient populations does not allow us to conclude that the framework will also perform as well when used on non-ideal patients.

### 3.4. Independent Variable Importance: The Clinical Signal Preserved by the Framework

**Figure 4:**
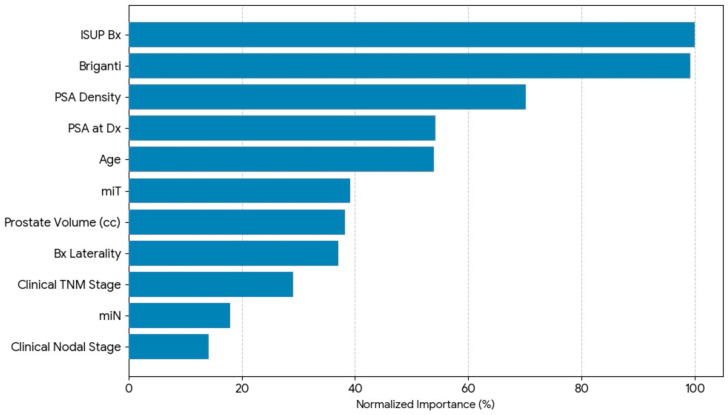
The normalised importance of clinical predictors in the 20/80 diagnostic model. The ISUP biopsy grade and the Briganti nomogram were identified as the primary drivers of risk stratification.

To evaluate the framework’s success in capturing and ranking clinical predictors, we examined the normalised predictive importance of each predictor for the 20/80 model using the decision-making process (Figure 4 and Table 6). The resulting ranking corresponds well with clinical evidence, confirming that the framework accurately captures (does not distort, and does not re-weight) the underlying medical reality.

The ISUP biopsy grade was the most important predictor, with a normalised predictive importance of 100%; thus, the ISUP biopsy grade is the gold standard for aggressiveness of PCa, and any credible model should put a higher priority on it. The second most important predictor was the Briganti nomogram, with a normalised predictive importance of 99.3%, which indicates that the framework accurately captured the complex, multifactorial signal found in this validated scoring system.

Significant weight was also given to more detailed biological markers. Compared to the absolute PSA value of 54.2%, PSA density achieved 70.2% importance. This preference implies that the framework captured the type of integrated thinking that competent clinicians do intuitively by preserving subtle, derived biomarkers over raw measurements. Prostate volume (38.2%) and age (54.0%) both made moderately significant contributions, indicating their functions as contextual risk modifiers.

Notably, modern multiparametric imaging data (mrT) were weighted 39.1%, while traditional clinical staging parameters were weighted the lowest: clinical TNM stage (29.1%), mrN (17.9%), and clinical nodal stage (14.1%). This hierarchy implies that the framework gave more weight to contemporary, high-information inputs than to earlier, coarser categorical descriptors, paralleling the continuing trend of evolving clinical practice toward more precise, multiparametric approaches.

The high value of the Briganti nomogram (99.3%) in comparison to the low value of the raw nodal stage (14.1%) is significantly different. The nodal status is one of the components of the Briganti nomogram, along with other variables. Therefore, the similarity of the nomogram to the ISUP grade suggests that it still serves as a “composite signal”—a probabilistically calculated combination of multiple risk factors that captures disease variation more completely than does any single categorical variable. This observation further supports the idea that the MLP should not replace confirmed clinical instruments, but instead adds an additional layer of refinement by utilising the accumulated knowledge from established clinical instruments while creating opportunities for non-linear integration of those findings.

The variable importance hierarchy, taken collectively, shows that the DQG design has retained a structure that is valid for the clinical environment. The model learned what it was supposed to learn: histology’s relative importance, that integrated value adds significant predictive power, that PSA density refines raw PSA score, and that modern imaging adds value to the traditional staging process. The fact that the framework aligned with the respective clinical expectations confirms that it can curate clinical data without creating any distortion.

The technical reports that contain detailed information for each model, including the complete case processing logs and raw parameter estimates, are available in the Supplementary Materials Files S4–S6 to support the transparency of the metrics that have been reported.

### 3.5. Comparative Benchmark: Evidence for Non-Linear Signal Preservation

We compared MLP performance with the gold standard, the exact logistic regression model (LogXact-11, Cytel Inc., Waltham, MA, USA), to determine if the DQG framework preserved clinically relevant non-linear relationships that traditional statistics may overlook [54]. The baseline model used five top-ranked MLP predictors (ISUP grade, Briganti nomogram, PSA density, age, and prostate volume) and was evaluated on the same 20/80 data split from which the MLP achieved perfect performance.

Table 7 demonstrates a striking difference in the performance of the logistic regression model. The overall model was significant (likelihood ratio *p* < 0.001), but it did not clearly show the significant individual predictors it used. Only ISUP created a statistically significant predictor independent of the model (*p* = 0.005), and neither of the two other predictors (Briganti, *p* = 0.800, and PSA density, *p* = 0.500) had enough evidence to mean that they were independent predictors of the model, even though when ranked by MLP, they should have been substantially equally predictive to ISUP. The age variable had a degenerate estimate (DEGEN), indicating that it exhibited quasi-complete separation and mathematical instability when using high-dimensional signals and small samples to produce its linear model [73,74,75].

The difference between these two models is quite helpful. For example, logistic regression (which is a linear model) could only find additive direct associations, although it could detect the ISUP grade, but could not see the additional predictive value from the Briganti nomogram or PSA density. In contrast, the MLP was able to integrate all these variables and assign the Briganti nomogram equal importance (99.3%) to that of the ISUP grade, as reflected by the 100% accuracy of the model at testing.

Taken together, these findings suggest that the MLP did not manufacture predictive power but rather exploited non-linear relationships (e.g., the modulation of PSA density by prostate volume and the composite structure of the Briganti nomogram) that the linear estimator could not capture under the present sample-size regime. Accordingly, the benchmark should be read not as a competition between methodologies but as evidence that the DQG framework preserves the full dimensionality of the clinical signal, leaving the MLP as the instrument through which that dimensionality becomes legible. Two caveats, however, must qualify this interpretation. First, exact logistic regression—like all parametric models—is known to become unstable in high-dimensional, small-sample settings, producing degenerate estimates (as observed here for the age variable) and inflating the standard errors of otherwise informative predictors; the non-significance of Briganti and PSA density in the linear model may, therefore, reflect small-sample instability of the parametric estimator rather than genuine absence of signal. Second, the apparently larger performance of the MLP may itself be partly attributable to overfitting on only 34 training cases (see Section 4.1, overparameterization argument). Accordingly, the contrast between the two models in Table 7 should be read as evidence that the DQG framework preserves a non-linear structure that is invisible to a linear estimator under the present sample-size regime, and not as a definitive demonstration of a superior non-linear signal-capture capability of the MLP per se.

## 4. Discussion

Before turning to the specific findings, we make explicit the analytical distinction that frames the entirety of this section, since it conditions the interpretation of every metric and limitation discussed below. Two evaluative axes coexist in this manuscript and must be kept analytically separate: (i) framework evaluation—assessing the FAIR-based data quality governance (DQG) protocol on its own merits as a reproducible, transferable and clinically coherent procedure for producing “AI-ready” urological datasets, and (ii) model performance evaluation—assessing the discrimination, calibration and clinical utility of the resulting multilayer perceptron (MLP). The present study provides admissible evidence for the former and explicitly inadmissible evidence for the latter. The AUC of 1.000, the 100% accuracy reported on the refined N = 9 test subset, and the variable-importance hierarchy reported below are illustrative by-products of applying the framework to a small single-centre cohort: they document that the framework yields a clinically coherent and learnable signal, but they cannot—and are not intended to—support claims of generalisable clinical performance. The reader is therefore invited to evaluate the framework, not the metrics, when judging the contribution of this work. The metrics are presented only insofar as they constitute auditable evidence that the framework operated as intended within its acknowledged validation constraints, and the multi-institutional pathway specified in Section Pre-Specified Sample-Size Justification and Three-Phase Multi-Institutional Validation Plan is the route through which the model itself—as opposed to the framework—would become eligible for performance evaluation.

This proof-of-concept study had two aims: first, to ascertain whether a DQG framework could empower a small, single-institutional dataset to generate a reliable predictive signal for PCa risk stratification through the application of an ANN, and second, to argue that the governance structure, rather than the resultant performance metrics, represents the fundamental contribution. This study’s outcomes substantiate the practicability of the initial objective, while also providing compelling evidence for the enduring significance of prioritising the latter.

### 4.1. The Fragility of Perfect Metrics in a Small-Sample Context

The 20/80 partition model achieved 100% testing accuracy (95% CI: 66.4–100%) and an AUC of 1.000 for discriminating between D’Amico high- and intermediate-risk patients. While mathematically valid, these statistics are best understood as an illustration of what this framework could achieve under idealised conditions rather than evidence of generalizable clinical superiority. Three interdependent methodological factors could explain this fragility.

First—and we now state this as the single most important limitation of the modelling approach, not as a secondary methodological note—the test set was not an independent hold-out sample but a refined validation cohort. The nine patients evaluated in the 20/80 configuration were not selected at random from the prostate cancer population; they were the residual cases left after the SPSS MLP framework automatically removed, at validation time, every test instance whose categorical factor levels (e.g., specific mrT/mrN stages or extreme PSA values) had not been represented in the training sample (Section 2.1.2 and Section Methodological Note and Limitation; Figure 1; Table 2). As a direct consequence, the patients most likely to stress any real prostate cancer model—clinical outliers, rare phenotypes, atypical presentations and extreme laboratory values—were systematically absent from the evaluation set. This is not an incidental feature of the software; it is a structural property of the present validation strategy: performance metrics computed on this refined subset therefore describe the behaviour of the model within the boundaries of the training distribution, and tell us essentially nothing about its behaviour outside those boundaries. Any claim of clinical generalisability based on these metrics would be epistemically unsound, and we explicitly make no such claim [76,77,78,79,80,81].

Second, there is a significant overparameterization of the model. Just 34 training samples were fitted to about 400 trainable parameters, so the parameters-to-samples ratio exceeded 10:1, a textbook high-dimensional setting with extreme risk of overfitting and dataset memorisation rather than pattern learning [82,83,84,85,86]. While the specific architectural choices employed, such as utilising a singular hidden layer to function as an information bottleneck [87,88,89,90,91] and implementing stringent early termination criteria, may have attenuated this potential risk, they are insufficient to entirely obviate it. The observed consistency in performance across diverse data subsets offers a measure of reassurance; however, this does not definitively preclude the possibility that the model merely assimilated peculiarities inherent to each specific partition, rather than genuinely deriving broadly applicable clinical principles.

Third, a major issue is the level of statistical uncertainty. The broad 95% confidence interval for the model’s predictive accuracy (66.4–100%) demonstrates the instability inherent in validating models using smaller data sets. Furthermore, even an isolated misclassification event among an independent validation cohort would profoundly impact the observed performance measures.

Accordingly, this raises an important implication: the apparent perfection of the model’s performance may have resulted from the meticulously controlled DQG framework from which it emerged; therefore, although its methodology is sufficient to reveal significant trends in structured data, it does not provide sufficiently conclusive evidence that its methodology can yield an instantiation of the model with sufficient robustness or generalisability for use within clinical practice. The results thus represent primarily a base for generating new hypotheses rather than providing sufficient empirical justification to implement changes in clinical protocols. This is why, throughout the rest of the Discussion and in the Conclusions, the deliverable we put forward for external scrutiny is the data quality governance framework itself, not the AUC of 1.000 or any other figure computed on the refined N = 9 test subset.

### 4.2. Evidence for an “AI-Premium”: Signal Detection or Overfitting?

The question of whether MLP has a genuine performance advantage compared to traditional models or simply illustrates its potential for overfitting remains to be answered [92,93,94,95]. We have found many instances of this throughout our comparison of exact logistic regression (Table 7).

The linear model, however, was positively significant (likelihood ratio *p* < 0.001), but only the ISUP grade was identified as an independent predictor (*p* = 0.005), whereas both the Briganti nomogram (*p* = 0.800) and PSA density (*p* = 0.500), both of which carry very high weights in the MLP model, were not. In fact, age had a degenerate parameter estimate (DEGEN) indicative of nearly complete separation [73,74,75]. In sharp contrast, the MLP combined all five of the most significant predictors to achieve a perfect accuracy rate on testing.

Two competing interpretations must be held in balance. The first posits that the MLP captured genuine non-linear synergies—such as the modulation of PSA density by prostate volume and the composite signal of the Briganti nomogram—that the linear estimator could not resolve, with the hidden layer acting as a refinement processor for the high/intermediate transition [51,52,53,96].

The second attributes the MLP’s superiority to overfitting on only 34 training cases, exploiting idiosyncrasies that the more conservative logistic regression correctly ignored. The truth likely lies between these poles: some non-linear signal was probably captured, but a component of overfitting is unavoidable at this sample size. The “AI-premium” observed here should, therefore, be treated as a hypothesis for external validation, not an established finding.

### 4.3. Clinical Significance of Predictive Variables

The analysis of variable importance (Figure 3; Table 6) indicates that the DQG framework preserved clinically significant associations rather than introducing any misrepresentations. The ISUP biopsy grade notably emerged as the paramount predictor, demonstrating maximal predictive power within the model, which is fitting since it serves as the histopathological gold standard for assessing malignancy aggressiveness [97,98,99].

Notably, the Briganti nomogram demonstrated paramount significance, contributing 99.3% to the model’s decisions and substantially surpassing the individual importance of its constituent elements, such as the raw clinical nodal stage (14.1%). This “composite signal” phenomenon indicates that the MLP recognised the nomogram as a probabilistically integrated summary of multifactorial risk [64,65,66]. This allowed it to capture disease heterogeneity more comprehensively than any one categorical variable. Furthermore, the network also learned to use PSA density (70.2%) as a contextual modifier to replace absolute PSA (54.2%), which is consistent with recent evidence that PSA density discriminates better than absolute PSA [53].

Multiparametric imaging (mrT) has demonstrated substantial predictive power (39.1%), while traditional clinical stages have contributed the least (cTNM, 29.1%; nodal stage, 14.1%). As such, this observed hierarchy is congruent with evolving clinical paradigms that increasingly prioritise integrated, information-rich diagnostic inputs [36,38].

Overall, these findings show that the developed framework was able to curate the data without any distortions. The model learned the appropriate hierarchy of histology, composites of validated composite tools, and was derived from biomarker refinements as opposed to measuring [92,93,94,95]. These outcomes support the equivalence of the developed framework for capturing genuine signal data rather than generating any signal indicators [95].

### 4.4. The Potential Added Value of the Artificial Neural Network over Existing Nomograms

A legitimate concern when introducing any machine-learning tool is whether it offers tangible advantages over the nomograms that currently constitute the standard of care. In the present work, the MLP neither reduces the number of variables required per patient, since it is explicitly designed to operate on the same clinical inputs that already feed the D’Amico classification and the Briganti nomogram, nor does it claim superior discrimination in absolute terms, given that the reported AUC of 1.000 reflects a best-case scenario rather than a generalisable performance metric. We, therefore, do not position this ANN as a replacement for validated nomograms, but as a complementary layer whose potential value lies in three specific dimensions that linear tools cannot deliver.

First, the MLP is intended to function as a non-linear refinement layer for the so-called “grey zone” between intermediate and high risk [39,40,41,42], where linear nomograms are known to underperform. The benchmark against exact logistic regression (Table 7) illustrates this point: although the linear model identified the ISUP grade as a significant predictor, it failed to assign statistical weight to Briganti and PSA density, and produced a degenerate estimate for age, all of which the MLP successfully integrated. This suggests that the added value of the network is not incremental accuracy on easy cases, but the preservation of non-linear interactions (e.g., the modulation of PSA density by prostate volume, or the composite structure of the Briganti score) that are systematically lost when the same variables are entered into a linear model.

Second, the ANN is designed to deliver probabilistic, individualised risk estimates rather than categorical thresholds. Current nomograms return a point estimate that is subsequently forced into a three-tier D’Amico category, whereas the MLP produces a continuous probability that can be coupled with a prediction-level confidence interval. In scenarios such as multidisciplinary tumour boards, this richer output can support the identification of apparent high-risk patients whose non-linear signal suggests a biologically less aggressive disease (mitigating overtreatment) and of apparent intermediate-risk patients with occult aggressive features (triggering further staging with PSMA-PET) [93,94,95].

Third, and central to the proof-of-concept presented here, the ANN serves as a demonstrator for the data quality governance framework itself. The added value of this study is, therefore, methodological rather than algorithmic: it illustrates that, when a FAIR-compliant DQG protocol is applied, even a small single-centre cohort can yield a stable, interpretable, and clinically coherent variable-importance hierarchy [23,24,25,56,57,58]. In this sense, the ANN is the vehicle through which the value of the framework becomes measurable, not the end product itself.

Accordingly, the present work should not be read as a claim that ANNs outperform standard-of-care nomograms on a per-patient basis with the current evidence, but as a methodological demonstration that rigorous data governance enables non-linear architectures to recover clinical complexity that linear tools discard. Demonstrating a clinically meaningful improvement in discrimination, calibration, or net benefit over standard nomograms is explicitly deferred to the external-validation phase described in Section 4.6 (Limitations and Future Directions). The reader is, therefore, asked to separate two evaluative axes throughout this manuscript: (a) the evaluation of the framework—its reproducibility, its ability to preserve clinically coherent variable-importance hierarchies, and its transferability to other cohorts—which we submit for scrutiny as the actual scientific claim of this work, and (b) the evaluation of the model—its discrimination, calibration, and net clinical benefit—for which the present study provides no admissible evidence, given the refined N = 9 test subset, and which must await the multi-centre validation pathway outlined in Section 4.6.

It is also important to distinguish the scope of the present proposal from that of recent image-based deep learning models applied to mpMRI, such as the PCa-Mamba [55] or the multi-centre ensemble by Alzate-Grisales [56]. Those models address a fundamentally different clinical task—voxel-level detection and localisation of clinically significant PCa lesions from T2-weighted, diffusion-weighted, and DCE-MRI sequences—and, therefore, require large multi-centre imaging cohorts, pharmacokinetic modelling of contrast kinetics, and GPU-intensive spatiotemporal architectures. An intermediate, and in our view particularly instructive, position is illustrated by ProMT-ML, a deployed machine-learning triage model that uses only four routinely available clinical variables (age, PSA, body mass index, and either prostate volume or systolic blood pressure) to estimate the probability of an abnormal prostate MRI, and outperforms PSA-based triage across more than 11,000 retrospective scans and 4500 prospective cases [100]. In contrast, the MLP presented here operates on eleven pre-engineered clinical, biochemical, and imaging-derived variables—including the categorical mrT/mrN descriptors and the Briganti nomogram score—and its purpose is post-detection D’Amico risk stratification rather than lesion detection or imaging triage. The three families of models are, therefore, complementary rather than competing: image-based pipelines, such as PCa-Mamba, could, in principle, provide upstream refined imaging-derived inputs to a downstream tabular risk-stratification layer of the kind explored in this study, while tabular triage models, such as ProMT-ML, could regulate which patients enter the imaging pipeline in the first place. Moreover, the methodological contribution of the present work—a FAIR-compliant data quality governance protocol that is agnostic to model architecture—is directly transferable to the curation of the structured metadata that accompanies image-based cohorts, and is not tied to any specific deep-learning backbone.

#### Positioning of the Proposed MLP Within the Existing Risk-Stratification Landscape

To make explicit how the present model relates to the principal families of PCa risk-stratification tools currently described in the literature, we summarise five representative comparators across the dimensions most relevant for clinical interpretation (Table 8): input modality, primary clinical task, approximate development sample size, data-quality safeguards reported by the original authors, and the principal claim made for each tool. The MLP described here is positioned not as a competitor to image-based deep-learning pipelines or to large, externally validated nomograms, but as a tabular, post-detection D’Amico risk-stratification layer whose distinctive contribution is the FAIR-based DQG protocol that enables non-linear modelling on a small single-centre cohort.

Two points emerge from this comparison, and we wish to make them unambiguous. First, the proposed MLP is not competing on sample size, AUC, or breadth of inputs: it is competing on data-quality discipline. None of the comparator approaches reports a comparably explicit, ISO-aligned, FAIR-based DQG protocol with a dedicated AI-readiness SOP, dual-blinded extraction with consensus reconciliation, and pre-specified operational validation rules at the level of detail provided in Table 1 and Appendix A. Second, the three families of comparators (linear nomograms, image-based deep-learning, tabular triage models) operate at different points of the diagnostic pathway and are mutually complementary rather than substitutable; the framework presented here is, by design, agnostic to where in that pathway it is applied, and could in principle be transferred without modification to the curation of the structured metadata that accompanies image-based cohorts or to the development of larger tabular triage models.

### 4.5. Data Quality as a Strategic Imperative

Even with a limited participant pool (N = 49), the integrity of this study’s findings was ensured by stringent preparatory measures designed for algorithmic analysis. This involved systematic data refinement, internal coherence verifications, and the judicious exclusion of incomplete or biologically implausible cases, thereby mitigating extraneous variation and fostering stable model performance. Compliance with the FAIR principles (Findable, Accessible, Interoperable, Reusable) directly confronts the contemporary challenge of reproducibility in medical AI [58,59,60,67]. This adherence guarantees that research datasets are not merely accessible but are also robustly structured for reliable algorithmic processing.

The availability of model weights using PMML/XML [102,103,104] provides transparency; however, the documentation of methods used to prepare the data (AI-Readiness Standard Operating Procedures and Data Quality Governance Checklist, Appendix A) is just as important in providing independent validation of the model and data curating logic for AI-assisted clinical decision support systems.

While the provision of model parameters in formats such as PMML/XML [102,103,104] enhances transparency, comprehensive documentation of the data preparation methodologies (e.g., preprocessing, feature coding, and/or normalisation) is of comparable importance. This documentation has been included in both the AI-Readiness SOPs and the Data Quality Governance Checklist (Supplemental Material File S1; Appendix A). Such detailed documentation facilitates independent scrutiny of both the analytical model and its underlying data curation logic, thereby cultivating confidence in AI-powered decision support frameworks.

Building on these methodological steps, we propose a staged development roadmap designed to explicitly meet the stated objectives of this proof-of-concept study and to address the fragility of the current metrics.

Stage 1 should focus on expanding the cohort to include a sufficient number of low-risk patients so that the model can be retrained as a three-class classifier aligned with the full D’Amico stratification; simulation-based power calculations suggest that a minimum of 150–200 additional cases, with balanced class representation, would be required to mitigate the overfitting risk that precluded the inclusion of the low-risk output in the present work [82,83,84,85,86].

Stage 2 should implement one-hot encoding of all categorical predictors prior to data partitioning, eliminating the automatic exclusion of rare factor levels and enabling an unbiased evaluation of the model on a fully representative hold-out set [76,77,78,79,80,81].

Stage 3 should incorporate uncertainty quantification through bootstrap resampling and SHAP-based attribution, producing confidence intervals for both performance metrics and variable importance rankings.

Stage 4 should carry out multi-centre external validation, using prospective or pre-registered retrospective cohorts, with pre-specified reporting of subgroup performance and calibration metrics in addition to discrimination [77,78].

Recent multi-institutional radiomics work illustrates the feasibility and the expected ceiling of this step [105], combining habitat-based and peritumoral radiomics from mpMRI across three centres and 896 patients, reaching external-validation AUCs of 0.860–0.876 for preoperative high-risk PCa prediction—a realistic performance target that is considerably below the AUC of 1.000 reported on our small single-centre sample and, therefore, underscores, rather than contradicts, the need to re-test the present framework on comparably large, heterogeneous cohorts.

Only after the completion of Stage 4 would the model be eligible for benchmarking against standard nomograms in terms of net clinical benefit (e.g., decision-curve analysis) and for consideration as a candidate tool for clinical deployment under the safeguards described in Section 4.7.

This commitment to reproducible, auditable reporting is aligned with the recently updated TRIPOD + AI statement, which replaces the original TRIPOD guideline to explicitly address the methodological and reporting particularities of regression- and AI/ML-based clinical prediction models, together with its dedicated adherence-assessment tool [106]. Prospective compliance with TRIPOD + AI—covering model development, model evaluation, or both—should be regarded as an integral part of any future iteration of the framework proposed here, and as a minimum standard for the external-validation roadmap outlined in Section 4.6.

#### Pre-Specified Sample-Size Justification and Three-Phase Multi-Institutional Validation Plan

The cohort-expansion target referenced in Stage 1 of the roadmap above (~150–200 additional cases) is here operationalised, in response to the sample-size and external-validation concerns that constitute the principal limitations of the present work. We adopt a deliberately conservative target derived from established recommendations for clinical prediction models in the small-sample regime, taking ten events per candidate predictor (10 EPV) for the rarest class as a minimum floor [82,83,84,85,86,107]. With 11 candidate predictors entering the network, this implies a floor of approximately 110 events in the minority D’Amico class for stable estimation [108,109,110]. Since high-risk cases account for approximately 50% of incident PCa in our institutional registry, this translates into a minimum recruitment target of approximately 220 evaluable patients to enable a stable binary high-vs-intermediate classifier. To enable the three-class (low/intermediate/high) classifier that the present study could not deliver—recall that the low-risk output had to be dropped from the 20/80 partition because of underrepresentation—and assuming a 10–15% prevalence for low-risk in screened cohorts, the recruitment floor rises to approximately 700–1100 evaluable patients. These figures are intentionally conservative and ignore additional considerations, such as class-balance under stratified splitting, calibration precision, and the higher EPV requirements that have been advocated for non-linear machine-learning estimators, which empirical reviews have shown to be substantially larger than those for regression-based approaches in oncology [82,83,84,85,86,111]; both will be re-estimated formally with simulation-based methods (e.g., the pmsampsize approach) once the Phase A cohort matures and the empirical event rate, predictor distributions, and outcome incidence have been measured, rather than assumed. Building on this floor, we pre-specify a three-phase multi-institutional validation pathway, summarised in Table 9:

Phase A—Single-centre cohort expansion (Clínica Universidad de Navarra, Pamplona). Expansion of the index cohort to ≥220 evaluable patients (≥110 high-risk events) at the original development site, retaining the FAIR-based DQG protocol and AI-Readiness SOPs described in this manuscript without modification. This phase has four purposes: (i) to replenish the low-risk stratum so that a three-class classifier becomes feasible; (ii) to test whether the framework scales without methodological drift when applied to a larger contemporaneous cohort at the same site; (iii) to enable a methodologically rigorous three-way partitioning into training, validation and hold-out test subsets—which the present sample size precluded, and which is the principal reason the current work could not separate hyperparameter selection from final-model evaluation—so that architectural choices, learning-rate adjustment, and early-stopping decisions are made exclusively on a dedicated validation subset, leaving the test partition strictly untouched until the final evaluation step; and (iv) to support repeated stratified k-fold cross-validation as a complementary stability check on training-time variance.

Phase B—Within-system, between-site validation (Clínica Universidad de Navarra, Madrid). External validation on a geographically distinct but methodologically aligned second site within the same institutional system. Because the EHR architecture, imaging protocols, and pathology workflows are largely shared across the two sites, Phase B isolates the contribution of patient-population heterogeneity from that of data-curation heterogeneity, providing a controlled first test of the framework’s transferability. Pre-specified endpoints include discrimination (AUC), calibration (calibration intercept and slope, and Brier score), decision-curve analysis, and TRIPOD + AI-compliant subgroup performance reporting [105].

Phase C—Multi-institutional national validation (Spanish urology network). Final, prospective, or pre-registered retrospective validation across additional Spanish hospitals with heterogeneous EHR systems, imaging vendors, and pathology practices. This phase explicitly targets the principal threat to external validity—data-curation heterogeneity—and constitutes the critical test of the FAIR-based DQG framework’s portability beyond a single institutional ecosystem. Variation in case-mix, missingness patterns, and coding conventions is expected to be the strongest stressor of the framework and will be reported in dedicated subgroup analyses by site.

Only after successful completion of Phase C, with pre-specified TRIPOD + AI-compliant reporting [106], will the model be considered eligible for entry into the cautionary clinical-translation pathway outlined in Section 4.7. Before that point, neither the AUC, the calibration metrics, nor the variable-importance hierarchy reported in this manuscript should be regarded as performance evidence; they remain, throughout, evidence about the framework, not about the model.

Across all three phases, prospective safeguards against data leakage will be pre-specified and audited as a formal extension of the DQG protocol. These safeguards include: (i) derivation of all feature-scaling parameters (means, standard deviations, and min–max normalisation bounds) exclusively from the training partition, with the same fitted parameters subsequently applied—without re-estimation—to the validation and test partitions; (ii) the fitting of any missing-data imputation procedure on training data only, with the resulting model frozen before being applied to the held-out subsets; (iii) patient-level identifier tracking across partitions to ensure that no individual patient contributes records to more than one subset; (iv) the explicit exclusion of any candidate predictor whose value would not have been available at the intended decision-time of the model in clinical practice; and (v) pre-registration of the analysis plan, including the partitioning scheme and all hyperparameter ranges, before the test partition is unsealed. These procedures are routine in canonical machine-learning practice but, as recent methodological reviews of medical AI have documented, are violated with sufficient frequency in published studies to constitute a recognised threat to replicability and the principal driver of the gap between reported and deployed performance [112,113,114]. Their explicit incorporation into the DQG protocol is, therefore, not a methodological refinement but a precondition for the framework’s claim to AI-readiness.

### 4.6. Limitations and Future Directions

As indicated earlier, a number of limitations cannot be dismissed, including: (a) the small sample size (N = 49), based on a strict curation and not *a priori* power analysis, coupled with wide confidence intervals, suggests substantial statistical instability in the data; (b) selection bias due to the exclusion of subjects with unusual factor levels (although mathematically required given the constraints of the presently available software), introduces further uncertainty about how well the model will perform on those rare phenotypic presentations; (c) that the importance is reported from single runs of the analysis and not based on bootstrap confidence intervals, prohibits the ability to quantify uncertainty surrounding feature rankings; (d) the fixed random seed (2,000,000), while allowing the replicability of our findings but constraining the full exploration of the model’s intrinsic stochastic variability.

Future research should adhere to a prescriptive pathway. Firstly, the model requires external validation across numerous multi-site cohorts that include the full spectrum of disease, including those with clinical characteristics that were excluded from this study [76,77,78,79,80,81]. Secondly, all categorical variables should be converted into a “one-hot-encoded” format before partitioning, so that all hold-out samples can be evaluated against a sample from which they were selected. Thirdly, an objective evaluation of how the model predicted outcomes relative to logistic regression models using just ISUP and Briganti should be conducted. Fourthly, the recruitment of additional subjects with low risk could provide opportunities to validate the findings of the D’Amico classification model for different levels of risk. Fifthly, methods to quantify uncertainty around how each predictor impacts prediction should be developed using either bootstrap resampling methods or SHAP-based methods. Lastly, multiple random seed-based analyses should be conducted to provide confidence intervals around the performance metrics.

### 4.7. Clinical Translation: A Cautionary Framework, Not a Deployable Tool

The restrictions mentioned above show that this model is not currently prepared for, and may never be prepared for, direct patient contact. The methodology developed in this research provides a guide to developing responsible approaches to moving AI technologies from proof-of-concept to clinical application. This guide also serves as a formal benchmark for assessing subsequent research in this area.

Before any application of clinical information could be made, a stable governance framework, with one or more models, must be validated by large cohorts from multiple institutions, reflecting the whole spectrum of the disease, including phenotypes that were systematically excluded, as well as clinical outliers [76,77,78,79,80,81]. Such validation requires either prospective studies or carefully designed retrospective studies that are designed using a variety of electronic health record (EHR) systems and that have pre-registered analyses. In addition, performance deteriorating across subgroups should be reported transparently [77,78].

Should its validation proceed as anticipated, the MLP is a candidate for use as a deployment model. When generalisability has been established through future work, the MLP could serve as a “digital second opinion” during multi-disciplinary tumour case review meetings, meaning it would be complementary to, not a replacement for, clinical judgment. Specifically, the MLP would be able to provide guidance in those clinical situations when there is uncertainty surrounding appropriate management of patients (e.g., “the grey zone” of clinical decision-making, as described by D’Amico and Briganti) [97,98,99]. The clinician could enter 11 variables routinely collected at the time of diagnosis into the MLP; in response, the MLP would generate a probability-based risk classification that may, for instance, identify an apparent high-risk patient whose non-linear signal indicates a biologically less aggressive cancer (thus avoiding overtreatment) or an apparent intermediate-risk patient with occult high-risk cancer features (thus triggering further staging with PSMA-PET). The attached PMML/XML documentation [102,103,104] provides the means to hypothetically integrate the MLP into current decision support systems without the need for proprietary software.

Given the importance of these aspects in future deployments, the additional protection elements that must be included are: (1) on-going monitoring for data drift and spectrum bias [81]; (2) including explicit confidence intervals associated with each prediction so uncertainty is communicated; (3) inclusion of a human oversight requirement, such that the model is only an advisory tool; and (4) regular re-evaluation as the population and clinical practices continue to change.

There is no current evidence to suggest that this model meets any of the above elements. The only usefulness of this work is to show how to create and govern datasets from which we may someday derive clinically useful models when we have a sufficient total number of satisfactory samples and when those samples represent our population. Only the framework for developing and supporting the datasets will require external validation.

## 5. Conclusions

This study shows that strict, FAIR-based data quality governance can produce a clear, comprehensible clinical signal from a small cohort, allowing algorithmic development. But it also serves as a sobering warning: the same governance that generates this clean signal can also produce a validation sample that is so carefully selected that performance metrics (like the 100% accuracy reported here) become clinically meaningless but mathematically perfect due to necessary but strict exclusion criteria. Therefore, the deliverable of this work—the one the reader is asked to evaluate, reproduce, and, eventually, challenge—is the transparent, repeatable FAIR-based DQG framework itself. The MLP, the AUC of 1.000, and the associated variable-importance hierarchy are illustrative by-products of applying that framework to a small single-centre cohort; they are not deployable artefacts and, given the refined N = 9 test sample, cannot be treated as generalisable performance evidence.

Within these explicit boundaries, this proof-of-concept study should be considered successful in the specific sense defined at the outset: it establishes that a FAIR-compliant DQG protocol can be operationalised, is reproducible, and enables a non-linear model to recover a clinically coherent variable-importance hierarchy from a small, single-centre cohort. Conversely, this proof-of-concept study is explicitly not successful in establishing clinical superiority over existing nomograms, nor in producing a deployable algorithm; both of these endpoints are, by design, deferred to the multi-stage validation roadmap outlined in Section 4.6. Distinguishing these two senses of “success” is, in itself, one of the contributions of this work to the ongoing debate on reproducibility in medical AI [58,59].

This framework’s performance on large, unselected, multi-institutional populations that represent the complete heterogeneity of clinical practice will be the real test of any model it generates.

## Figures and Tables

**Table 2:** The clinical characterisation and frequency of the excluded subgroup across data partitioning schemes.

Partitioning Scheme	Total Sample	Valid Cases (n)	Excluded Cases (n)	Exclusion Rate (%)	Primary Reason for Exclusion
Model 20/80	49	43	6	12.20%	Factor levels (e.g., PSA values or mrT stages) not present in the training set.
Model 34/66	49	44	5	10.20%	Factor levels or dependent variable values (Low-risk strata) not present in training.
Model 39/61	49	41	8	16.30%	Factor levels (clinical outliers) not represented in the training sample.

**Table 3:** Model performance comparison.

Metric	Model 20/80	Model 34/66	Model 39/61
D’Amico Strata Evaluated	Binary (high/int)	Ternary (high/int/low)	Binary (high/int)
Training Sample n (%)	34 (79.10%)	29 (65.90%)	25 (61.00%)
Testing Sample n (%)	9 (20.9%)	15 (34.1%)	16 (39.0%)
Training Cross-Entropy Error	0.161	3.842	0.209
Testing Cross-Entropy Error	0.001	4.227	4.636
Training Incorrect Predictions (%)	0.00%	6.90%	0.00%
Testing Incorrect Predictions (%)	0.00%	13.30%	6.30%
Overall Training Accuracy (%)	100%	93.10%	100.00%
Overall Testing Accuracy (%)	100% (95% CI: 66.4–100) ^†^	86.70% (95% CI: 62.1–96.3)	93.80% (95% CI: 71.7–98.9)
Correct Classifications (n/N)	(9/9)	(13/15)	(15/16)
Sensitivity (High Risk)	100% (95% CI: 56.5–100)	85.7% (95% CI: 48.7–97.4)	87.5% (95% CI: 52.9–97.8)
Specificity (Int. Risk)	100% (95% CI: 51.0–100)	87.5% (95% CI: 52.9–97.8)	100% (95% CI: 67.6–100)
PPV (Positive Predictive Value)	100% (95% CI: 56.5–100)	85.7% (95% CI: 48.7–97.4)	100% (95% CI: 64.6–100)
NPV (Negative Predictive Value)	100% (95% CI: 51.0–100)	87.5% (95% CI: 52.9–97.8)	88.9% (95% CI: 56.5–98.0)

^†^ “Testing set confidence intervals for the 20/80 model were calculated using the Clopper–Pearson exact method in SPSS due to the small sample size (N = 9); all other intervals were calculated using the score method in OpenEpi.

**Table 4:** Area under the curve (AUC) comparison.

Risk Group	AUC Model 20/80	AUC Model 34/66	AUC Model 39/61
High	1	0.996	0.99
Intermediate	1	0.991	0.99
Low	N/A	1	N/A

**Table 5:** Classification matrix (20/80 model).

Sample	Observed Risk Group	Predicted: High	Predicted: Intermediate	Percent Correct
Training	High	22	0	100%
	Intermediate	0	12	100%
Testing	High	5	0	100%
	Intermediate	0	4	100%
Global Percentage		55.60%	44.40%	100%

**Table 6:** Independent variable importance (20/80 model).

Variable	Importance	Normalized Importance
ISUP BX	0.181	100.00%
BRIGANTI	0.180	99.30%
PSA DENSITY	0.127	70.20%
PSA at DX	0.098	54.20%
AGE	0.098	54.00%
mrT	0.071	39.10%
PROSTATE VOLUME c.c.	0.069	38.20%
BX LATERALITY	0.067	37.00%
Clinical TNM Stage	0.053	29.10%
mrN	0.032	17.90%
Nodal Stage	0.025	14.10%

**Table 7:** Comparative performance: multilayer perceptron (MLP) vs. exact logistic regression baseline.

Feature/Metric	Exact Logistic Regression (Baseline)	Multilayer Perceptron (MLP 20/80)
Statistical Engine	Exact Likelihood Estimation (LogXact).	Backpropagation/Softmax (SPSS).
Model Type	Linear/parametric.	Non-linear/connectionist + 1.
Overall Significance	*p* < 0.001 (likelihood ratio).	*p* = 0.002 (Exact Binomial Test).
Predictor: ISUP Grade	Significant (*p* = 0.005).	Dominant (100% importance).
Predictor: Briganti Nomogram	Not significant (*p* = 0.800).	Critical (99.3% importance).
Predictor: PSA Density	Not significant (*p* = 0.500).	High impact (70.2% importance).
Predictor: Age	Degenerate estimate (DEGEN). ^†^	Moderate impact (54.0% importance).
Testing Accuracy	Outperformed by MLP	100.00%.
Testing AUC	<1.000.	1.
Clinical Interpretation	Limited to linear histological signal.	Captures non-linear feature synergies.

^†^ Exact logistic regression performed via permutation method in LogXact-11 with N = 43 valid cases. DEGEN: degenerate estimate due to quasi-complete separation. ISUP: International Society of Urological Pathology; PSA: Prostate-Specific Antigen. MLP significance (*p* = 0.002) determined via One-Sample Exact Binomial Test.

**Table 8:** A comparison of the proposed MLP with representative existing PCa risk-stratification approaches.

Tool/ Approach	Input Data	Primary Clinical Task	Approximate Development Sample	Data-Quality Governance	Principal Claim
D’Amico classification [63]	3 clinical/biochemical variables	Pre-treatment risk stratification	Multi-cohort historical	Implicit (clinical staging conventions)	Validated three-tier risk groups
Briganti nomogram [64,65,66]	4–5 clinical/biochemical/pathological variables	Probability of lymph node invasion	>1000 patients (multi-cohort)	Standardised pathology review	Calibrated probabilistic estimate
Exact logistic regression (Table 7)	5 top MLP predictors	Linear risk discrimination	N = 43 valid cases (this study)	Same DQG protocol as MLP	Confirms ISUP signal; cannot integrate non-linear features
Image-based deep learning [101] (e.g., PCa-Mamba [55] multi-centre 3D EfficientNet [56])	mpMRI sequences (T2W, DWI, and DCE)	Lesion detection/csPCa	Thousands of MRI sessions, multi-centre	Variable; PI-CAI provides a curated benchmark	Lesion-level AUC ≈ 0.80–0.90
Tabular ML triage (e.g., ProMT-ML [100]	4 routinely available variables	Pre-MRI triage	>11,000 retrospective + 4500 prospective	Routine-care data; deployed pipeline	Outperforms PSA-based triage
Proposed MLP (this work)	11 clinical/biochemical/imaging-derived variables	Post-detection D’Amico risk stratification	N = 49 (proof-of-concept)	Explicit FAIR-based DQG with ISO/IEC 25012 alignment, dual blinded extraction, AI-readiness SOPs (Appendix A)	Methodological framework—not a deployable model

**Table 9:** Pre-specified three-phase multi-institutional validation roadmap.

Phase	Site (s)	Target Sample Size	Primary Objective	Pre-Specified Endpoints
A	CUN Pamplona (development site).	≥220 evaluable patients (≥110 high-risk events; replenished low-risk stratum).	Cohort expansion to enable three-class classifier and formal internal cross-validation.	Discrimination (AUC); calibration; bootstrap-based variable-importance CIs; SHAP attribution; documented anti-leakage safeguards (training-only fitting of scalers and imputers; patient-level partition integrity).
B	CUN Madrid (second site, same institutional system).	Independent cohort ≥150 evaluable patients.	Within-system external validation; isolation of population vs. curation heterogeneity.	AUC; calibration intercept/slope; Brier score; decision-curve analysis; TRIPOD + AI subgroup reporting; verification that anti-leakage safeguards from Phase A are preserved across sites.
C	Spanish multi-centre network (additional centres).	Multi-centre cohort ≥500 evaluable patients.	True multi-institutional external validation; test of framework portability across heterogeneous EHR/imaging/pathology ecosystems.	Site-stratified AUC and calibration; data-drift monitoring; net clinical benefit vs. standard nomograms; pre-registered analysis plan.

## Data Availability

The original contributions presented in this study are in the article. Further inquiries can be directed to the corresponding author.

## Supplementary Materials

The following supporting information can be downloaded at: https://www.mdpi.com/article/10.3390/diagnostics16101454/s1, File S1: The artificial neural network architecture in PMML/XML format for the 39/61 data partitioning scheme; File S2: The artificial neural network architecture in PMML/XML format for the 34/66 data partitioning scheme; File S3: The artificial neural network architecture in PMML/XML format for the 20/80 data partitioning scheme. File S4: The full SPSS Output Report for the 20/80 model, containing the original execution logs, comprehensive classification matrices, and raw neural weight estimates; File S5: The full SPSS Output Report for the 34/66 model, documenting the multi-category performance and factor level exclusions; File S6: The full SPSS Output Report for the 39/61 model, providing the complete model summary and variable importance data from the software execution.

## Abbreviations

The following abbreviations are used in this manuscript:

ANN	Artificial Neural Network
AUC	Area Under the Curve
cT	Clinical tumour stage
DQG	Data Quality Governance
DQG-AI	Data Quality Governance for AI-readiness framework
EHR	Electronic Health Record
FAIR	Findability, Accessibility, Interoperability, and Reusability
ISUP	International Society of Urological Pathology
mrN	Multiparametric imaging nodal stage (or Imaging nodal stage)
mrT	Multiparametric imaging tumour stage (or Imaging tumour stage)
MLP	Multilayer Perceptron
mpMRI	Multiparametric Magnetic Resonance Imaging
NPV	Negative Predictive Value
PCa	Prostate Cancer
PMML	Predictive Model Markup Language
PPV	Positive Predictive Value
PSA	Prostate-Specific Antigen
ROC	Receiver Operating Characteristic
SD	Standard Deviation
SHAPs	SHapley Additive exPlanations
SOP	Standard Operating Procedure
tanh	Hyperbolic Tangent
XML	Extensible Markup Language

## Appendix A. AI-Readiness and Data Quality Governance Checklist—DQG-AI Framework v1.0 (Clínica Universidad de Navarra)

Title: Standard Operating Procedure (SOP) for Clinical Data Curation in Artificial Neural Network Development.

This checklist outlines the minimum requirements used in this study to ensure that the dataset meets the criteria for “AI-readiness” before model training.

1. Data Integrity and Source Governance

[ ] Source Verification (Blinded): Every data point (PSA, ISUP, and mrTNM) was cross-referenced between the electronic health record (EHR) and the study database by two independent reviewers blinded to each other’s assessments.

[ ] Consensus Reconciliation: A formal reconciliation process was used to resolve any inter-rater discrepancies, ensuring 100% clinical concordance for all primary predictors before model training.

[ ] FAIR Compliance: Data fields were mapped to standard clinical vocabularies (e.g., TNM 8th Ed [115], ISUP 2014 [116]) to ensure interoperability and future reuse.

[ ] Anonymisation: All identifiers were removed in accordance with GDPR/Institutional Review Board standards prior to algorithmic processing.

2. Operational Data Quality Rules (The “Technical Gatekeeper”)

[ ] Range Constraint Validation: Continuous variables must fall within biological plausibility limits (e.g., PSA > 0.1; Age 40–90). Values outside these ranges were flagged for manual re-verification or exclusion.

[ ] Logical Consistency Checks: Staging hierarchies were preserved (e.g., a patient cannot be miN1 without clinical evidence of nodal involvement or suspicious imaging).

[ ] Missingness Protocol: A strict “complete-case analysis” was applied. Any record with missing data in any of the 11 primary clinical predictors was excluded to prevent “noise” in the backpropagation process.

3. Pre-Modelling “AI-Readiness” Transitions

[ ] Feature Encoding: All categorical variables (Factors) must use 1-of-c encoding (one-hot encoding) to ensure the network can mathematically distinguish between distinct clinical strata.

[ ] Scaling and Normalisation: Continuous covariates were rescaled using linear normalisation (min–max) to prevent gradient vanishing or weight saturation in the hidden layer’s activation functions (tanh).

[ ] Outlier Management: “Clinical outliers” (rare factor combinations) were documented and reported transparently, even if excluded by the software’s automatic validation protocol.

4. Evaluation and Stability Governance

[ ] Random Seed Fixation: A fixed random seed (e.g., 2,000,000) was used to ensure all training runs are reproducible by third parties.

[ ] Sensitivity Analysis: Model performance was evaluated across multiple data partitioning schemes (e.g., 80/20, 50/50) to ensure the reported accuracy is not an artefact of a single “lucky” split.

[ ] Uncertainty Reporting: Results were reported as distributions (Mean ± SD) rather than single peak-performance percentages.
