# Supplementary material for: Prioritising Data Quality Governance for AI in Prostate Cancer: A Methodological Proof-of-Concept Study Using Neural Networks for Risk Stratification
Source: Diagnostics (Basel). 2026 May 10;16(10):1454. doi: 10.3390/diagnostics16101454 (PMC13205476; doi:10.3390/diagnostics16101454)
Supplement: Supplementary file 1 [file diagnostics-16-01454-s001.zip › S4 OUTPUT39_61.pdf]

# Perceptrón multicapa

## Advertencias

| |
|---|
| Uno o más casos de la muestra de la prueba o la muestra reservada tienen un facto o valores de variable dependientes que no se producen en la muestra de entrenamiento. Estos casos se excluyen del análisis. |

## Resumen de procesamiento de casos

| | | N | Porcentaje |
|---|---|---|---|
| Ejemplo | Entrenamiento | 25 | 61,0% |
| | Pruebas | 16 | 39,0% |
| Válido | | 41 | 100,0% |
| Excluido | | 8 | |
| Total | | 49 | |

## Información de red

| | | | |
|---|---|---|---|
| Capa de entrada | Factores | 1 | PSA AL DX |
| | | 2 | ISUP BX |
| | | 3 | LATERALIDAD de BX |
| | | 4 | Estadio clínico segun TNM |
| | | 5 | Estadio clínico ganglionar |
| | | 6 | miT |
| | | 7 | miN |
| | Covariables | 1 | EDAD |
| | | 2 | DENSIDAD PSA |
| | | 3 | VOLUMEN PROSTATA |

| | | | |
|---|---|---|---|
| | | 4 | c.c. BRIGANTI |
| | Número de unidades[a] | | 41 |
| | Método de cambio de escala para las covariables | | Estandarizados |
| Capas ocultas | Número de capas ocultas | | 1 |
| | Número de unidades en la capa oculta 1[a] | | 6 |
| | Función de activación | | Tangente hiperbólica |
| Capa de salida | Variables dependientes | 1 | GRUPO DE RIESGO (D´amico) |
| | Número de unidades | | 2 |
| | Función de activación | | Softmax |
| | Función de error | | Entropía cruzada |

a. Se excluye la unidad de sesgo

## Resumen del modelo

| | | |
|---|---|---|
| Entrenamiento | Error de entropía cruzada | ,209 |
| | Porcentaje de pronósticos incorrectos | 0,0% |
| | Regla de parada utilizada | 1 paso(s) consecutivo(s) sin disminución del error[a] |
| | Tiempo de entrenamiento | 0:00:00,01 |
| Pruebas | Error de entropía cruzada | 4,636 |
| | Porcentaje de pronósticos incorrectos | 6,3% |

Variable dependiente: GRUPO DE RIESGO (D´amico)

a. Los cálculos de error se basan en la muestra de comprobación.

**Estimaciones de parámetro**

| Predictor | | Pronosticado | | | | | | | |
|---|---|---|---|---|---|---|---|---|---|
| | | Capa oculta 1 | | | | | | Capa de salida | |
| | | H(1:1) | H(1:2) | H(1:3) | H(1:4) | H(1:5) | H(1:6) | [GRUPODERIESGO=Alto] | [GRUPODERIESGO=Intermedio] |
| Capa de entrada | (Sesgo) | -,418 | -,079 | ,205 | ,660 | ,027 | -,006 | | |
| | [PSAALDX=2] | ,397 | -,477 | -,033 | ,067 | ,171 | ,128 | | |
| | [PSAALDX=4] | -,338 | -,071 | -,887 | -,229 | ,370 | -,233 | | |
| | [PSAALDX=5] | ,173 | -,024 | -,259 | ,541 | ,219 | ,042 | | |
| | [PSAALDX=6] | -,154 | ,082 | ,166 | -,431 | -,059 | ,269 | | |
| | [PSAALDX=7] | ,097 | -,199 | ,477 | ,174 | ,699 | -,213 | | |
| | [PSAALDX=8] | ,658 | ,088 | ,688 | -,318 | ,392 | -,056 | | |
| | [PSAALDX=9] | ,516 | ,386 | ,378 | -,129 | ,252 | -,227 | | |
| | [PSAALDX=10] | -,416 | -,347 | ,251 | ,096 | ,009 | ,502 | | |
| | [PSAALDX=11] | -,309 | ,168 | -,471 | ,012 | -,369 | -,266 | | |
| | [PSAALDX=12] | -,403 | -,285 | -,158 | ,271 | ,294 | -,323 | | |
| | [PSAALDX=13] | -,177 | -,664 | ,295 | -,076 | ,167 | -,287 | | |
| | [PSAALDX=16] | -,387 | -,198 | ,514 | -,234 | -,631 | -,560 | | |
| | [PSAALDX=19] | ,141 | ,350 | -,048 | ,183 | -,460 | -,377 | | |
| | [PSAALDX=20] | ,460 | ,876 | ,370 | ,141 | ,391 | -,215 | | |
| | [PSAALDX=48] | ,556 | ,431 | ,410 | ,464 | -,280 | ,441 | | |
| | [PSAALDX=69] | -,118 | ,071 | -,351 | ,282 | ,339 | ,129 | | |
| | [ISUPBX=1] | ,975 | ,301 | -1,021 | ,238 | ,087 | -,039 | | |
| | [ISUPBX=2] | ,598 | ,158 | -,185 | ,070 | ,220 | -,182 | | |
| | [ISUPBX=3] | ,204 | -,213 | -,576 | ,025 | -,571 | -,928 | | |
| | [ISUPBX=4] | -,091 | ,456 | ,798 | -,586 | ,666 | 1,099 | | |
| | [ISUPBX=5] | -,196 | -,262 | ,025 | -,408 | ,006 | ,452 | | |
| | [LATERALIDAD | -,129 | -,579 | -,307 | ,294 | ,089 | -,672 | | |

| | | | | | | | | | |
|---|---|---|---|---|---|---|---|---|---|
| | deBX=Bilateral] | | | | | | | | |
| | [LATERALIDAD deBX=Derecho ] | -,295 | ,175 | -,091 | ,858 | -,326 | -,083 | | |
| | [LATERALIDAD deBX=Izquierdo] | -,821 | -,299 | -,057 | -,114 | ,325 | -,358 | | |
| | [cT=T1c] | ,198 | -,354 | -,175 | -,055 | -,578 | ,116 | | |
| | [cT=T2a] | -,299 | -,711 | -,130 | ,069 | ,021 | -,286 | | |
| | [cT=T2b] | -,153 | -,323 | -,143 | ,291 | ,337 | ,289 | | |
| | [cT=T2c] | -,268 | ,008 | ,108 | -,288 | -,473 | ,488 | | |
| | [cT=T3a] | ,489 | ,616 | -,005 | ,157 | ,260 | ,054 | | |
| | [cN=N0] | ,114 | ,386 | ,290 | ,336 | ,587 | ,533 | | |
| | [cN=Nx] | -,476 | -,309 | ,392 | -,331 | -,368 | ,103 | | |
| | [miT=miT 2] | ,357 | ,305 | -,290 | -,445 | -,047 | -,056 | | |
| | [miT=miT 3] | ,024 | -,344 | -,304 | ,590 | -,235 | -,174 | | |
| | [miT=miT3a] | -,364 | ,398 | ,217 | ,053 | ,155 | ,449 | | |
| | [miT=miT3b] | -,159 | ,632 | ,160 | -,147 | ,373 | -,014 | | |
| | [miN=miN 0] | -,282 | ,062 | -,120 | -,269 | ,442 | ,112 | | |
| | [miN=miN 1] | ,527 | -,431 | ,178 | ,062 | ,149 | ,159 | | |
| | EDAD | ,311 | -,226 | ,017 | -,539 | ,520 | ,340 | | |
| | DENSIDADPSA | -,110 | ,327 | ,772 | -,376 | ,160 | ,368 | | |
| | VOLUMENPROSTATAc.c | -,021 | -,001 | ,325 | ,399 | -,006 | -,092 | | |
| | BRIGANTI | ,267 | -,298 | -,292 | -,595 | ,203 | ,675 | | |
| Capa oculta 1 | (Sesgo) | | | | | | | -,355 | -,254 |
| | H(1:1) | | | | | | | -,631 | ,061 |
| | H(1:2) | | | | | | | ,484 | -,104 |
| | H(1:3) | | | | | | | ,733 | -,889 |
| | H(1:4) | | | | | | | -,024 | ,989 |
| | H(1:5) | | | | | | | ,680 | -,816 |

| | | | | | | | | |
|---|---|---|---|---|---|---|---|---|
| H(1:6) | | | | | | | 1,640 | -1,627 |

## Clasificación

| Ejemplo | Observado | Pronosticado Alto | Pronosticado Intermedio | Pronosticado Porcentaje correcto |
|---|---|---|---|---|
| Entrenamiento | Alto | 15 | 0 | 100,0% |
| | Intermedio | 0 | 10 | 100,0% |
| | Porcentaje global | 60,0% | 40,0% | 100,0% |
| Pruebas | Alto | 7 | 1 | 87,5% |
| | Intermedio | 0 | 8 | 100,0% |
| | Porcentaje global | 43,8% | 56,3% | 93,8% |

Variable dependiente: GRUPO DE RIESGO (D´amico)

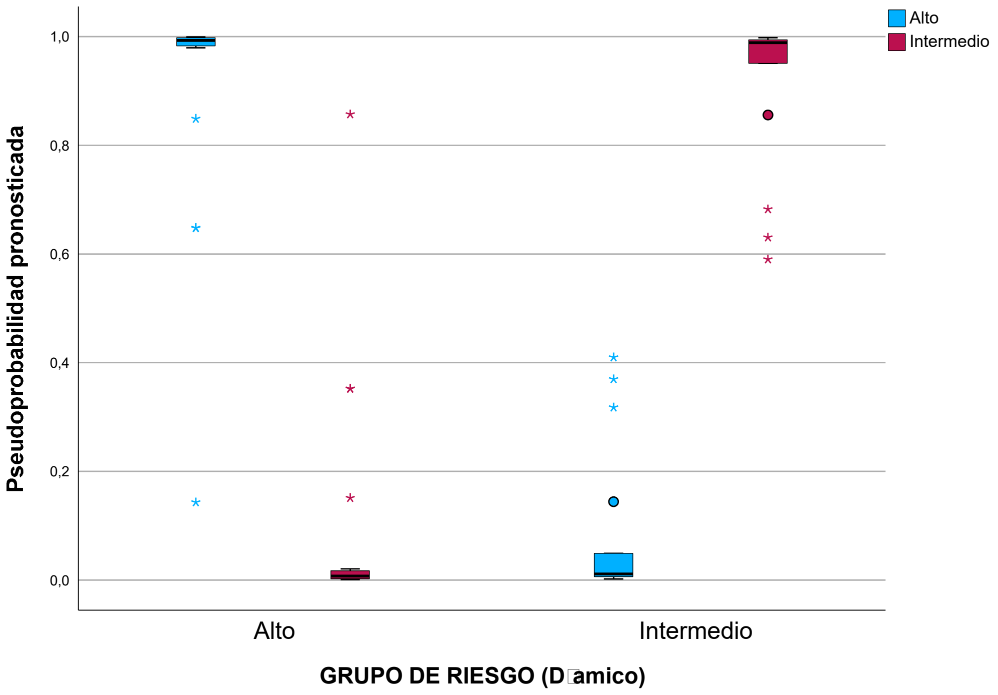

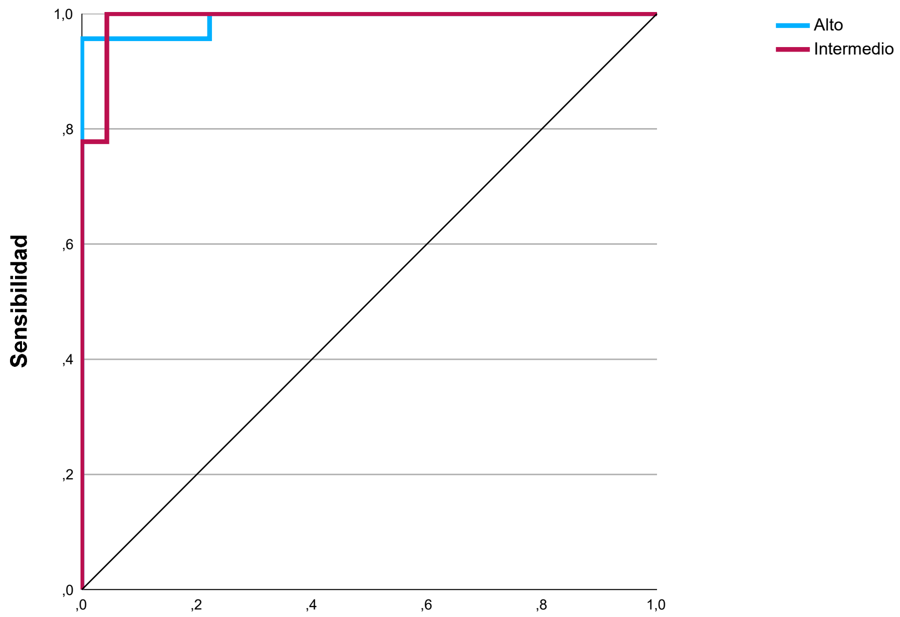


Variable dependiente: GRUPO DE RIESGO (D´amico)

## Área bajo la curva

| | | Áreas |
|---|---|---|
| GRUPO DE RIESGO (D´amico) | Alto | ,990 |
| | Intermedio | ,990 |

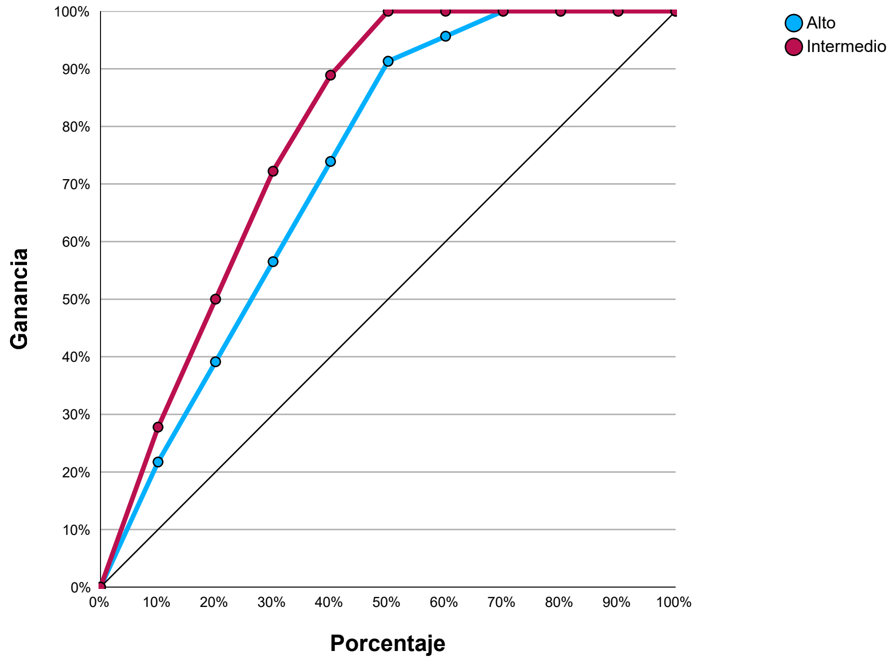


Variable dependiente: GRUPO DE RIESGO (D´amico)

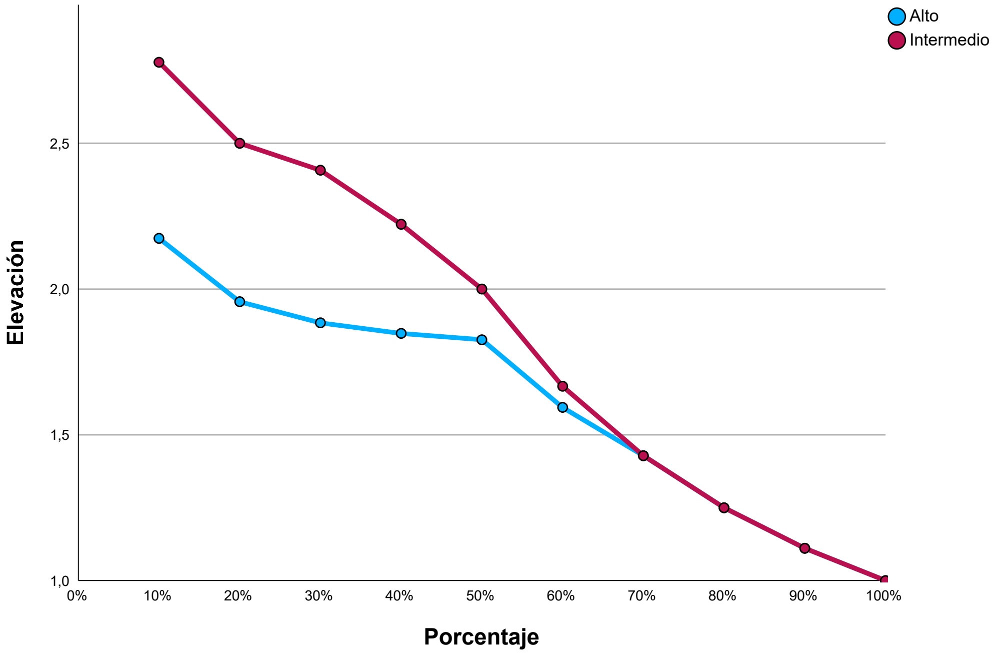


Variable dependiente: GRUPO DE RIESGO (D´amico)

## Importancia de las variables independientes

| | Importancia | Importancia normalizada |
|---|---|---|
| PSA AL DX | ,086 | 40,2% |
| ISUP BX | ,213 | 100,0% |
| LATERALIDAD de BX | ,050 | 23,3% |
| Estadio clínico segun TNM | ,057 | 26,7% |
| Estadio clínico ganglionar | ,038 | 18,0% |
| miT | ,081 | 37,8% |
| miN | ,020 | 9,2% |
| EDAD | ,139 | 65,4% |
| DENSIDAD PSA | ,171 | 80,2% |
| VOLUMEN PROSTATA c.c. | ,029 | 13,5% |
| BRIGANTI | ,117 | 54,9% |

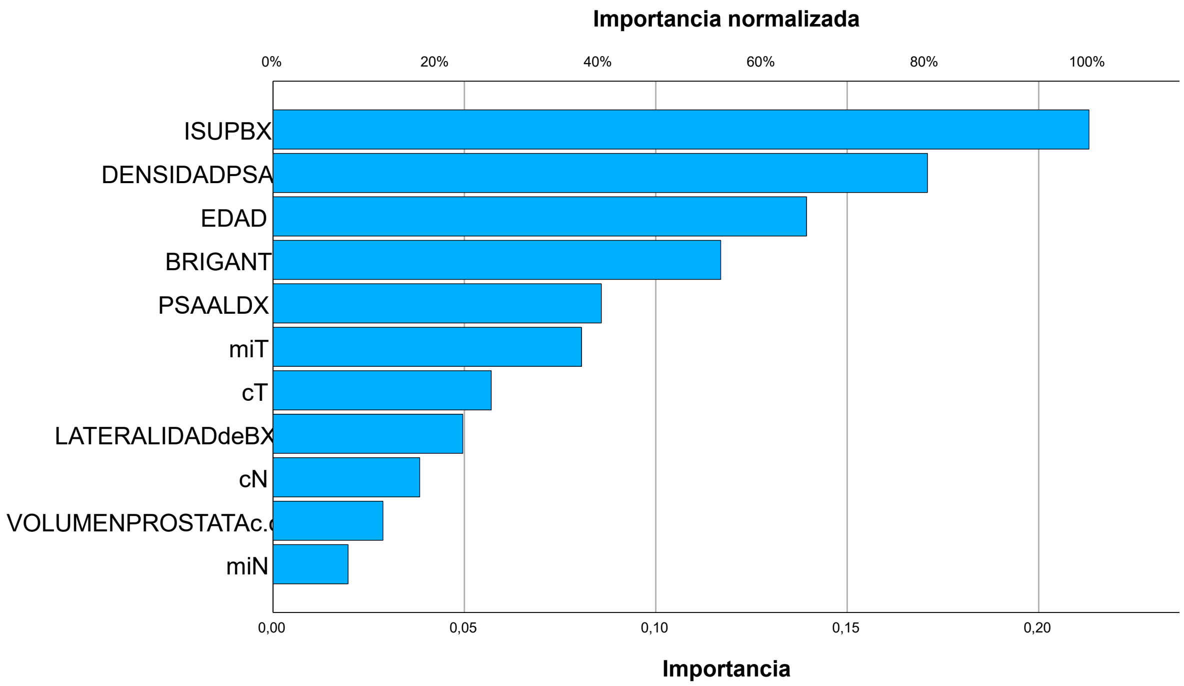
Importancia normalizada
0%
20%
40%
60%
80%
100%
ISUPBX
DENSIDADPSA
EDAD
BRIGANT
PSAALDX
miT
cT
LATERALIDADdeBX
cN
VOLUMENPROSTATAc.c
miN
0,00
0,05
0,10
0,15
0,20
Importancia
