# Supplementary material for: Prioritising Data Quality Governance for AI in Prostate Cancer: A Methodological Proof-of-Concept Study Using Neural Networks for Risk Stratification
Source: Diagnostics (Basel). 2026 May 10;16(10):1454. doi: 10.3390/diagnostics16101454 (PMC13205476; doi:10.3390/diagnostics16101454)
Supplement: Supplementary file 1 [file diagnostics-16-01454-s001.zip › S5 OUTPUT34_66.pdf]

Perceptrón multicapa

Advertencias

Uno o más casos de la muestra de la prueba o la muestra reservada tienen un facto o valores de variable dependientes que no se producen en la muestra de entrenamiento. Estos casos se excluyen del análisis.

Resumen de procesamiento de casos

		N	Porcentaje
Ejemplo	Entrenamiento	29	65,9%
	Pruebas	15	34,1%
Válido		44	100,0%
Excluido		5	
Total		49	

Información de red

Capa de entrada	Factores	1	PSA AL DX
		2	ISUP BX
		3	LATERALIDAD de BX
		4	Estadio clínico segun TNM
		5	Estadio clínico ganglionar
		6	miT
		7	miN
	Covariables	1	EDAD
		2	DENSIDAD PSA
		3	VOLUMEN PROSTATA

		4	c.c. BRIGANTI
	Número de unidades[a]		42
	Método de cambio de escala para las covariables		Estandarizados
Capas ocultas	Número de capas ocultas		1
	Número de unidades en la capa oculta 1[a]		6
	Función de activación		Tangente hiperbólica
Capa de salida	Variables dependientes	1	GRUPO DE RIESGO (D´amico)
	Número de unidades		3
	Función de activación		Softmax
	Función de error		Entropía cruzada

a. Se excluye la unidad de sesgo

Resumen del modelo

Entrenamiento	Error de entropía cruzada	3,842
	Porcentaje de pronósticos incorrectos	6,9%
	Regla de parada utilizada	1 paso(s) consecutivo(s) sin disminución del error[a]
	Tiempo de entrenamiento	0:00:00,04
Pruebas	Error de entropía cruzada	4,227
	Porcentaje de pronósticos incorrectos	13,3%

Variable dependiente: GRUPO DE RIESGO (D´amico)

a. Los cálculos de error se basan en la muestra de comprobación.

Estimaciones de parámetro

	Predictor	Pronosticado							
		Capa oculta 1						Capa de salida	
		H(1:1)	H(1:2)	H(1:3)	H(1:4)	H(1:5)	H(1:6)	[GRUPODERIESGO=Alto]	[GRUPODERIESGO=Bajo]
Capa de entrada	(Sesgo)	,191	,173	-,218	,203	,729	,220		
	[PSAALDX=2]	,232	,007	,050	,306	-,400	,058		
	[PSAALDX=4]	-,315	-,212	-,173	,571	,340	,018		
	[PSAALDX=5]	,278	,018	,212	-,396	,082	-,387		
	[PSAALDX=6]	-,134	-,342	,126	,199	,124	,078		
	[PSAALDX=7]	,322	-,304	-,482	-,309	,176	,174		
	[PSAALDX=8]	,166	-,147	,494	,273	-,439	-,269		
	[PSAALDX=9]	,021	-,487	-,055	-,152	-,225	,148		
	[PSAALDX=10]	,474	,433	-,028	,455	-,347	,124		
	[PSAALDX=11]	-,297	-,144	,425	,042	,421	,002		
	[PSAALDX=12]	,512	,159	-,351	,513	-,370	,338		
	[PSAALDX=13]	,141	,282	,539	,182	-,064	,331		
	[PSAALDX=16]	,381	,328	-,275	-,371	,217	,164		
	[PSAALDX=19]	,680	-,005	-,571	,195	-,318	,459		
	[PSAALDX=20]	-,257	-,264	,206	-,266	,497	,174		
	[PSAALDX=21]	-,404	,280	,159	,045	-,286	,270		
	[PSAALDX=22]	-,370	,117	,241	,208	-,190	,495		
	[PSAALDX=36]	-,188	-,309	,544	,181	-,322	,180		
	[ISUPBX=1]	,351	,186	-,104	-,010	-,556	,467		
	[ISUPBX=2]	-,093	,175	,306	,384	,356	-,134		
	[ISUPBX=3]	1,083	-,148	-,277	,204	,225	,421		
	[ISUPBX=4]	-1,234	,484	,556	-,905	,123	-,567		
	[ISUPBX=5]	-,144	,345	-,059	-,298	,263	-,325		
	[LATERALIDADdeBX=Bilateral]	-,023	,356	-,219	,064	-,230	,254		
	[LATERALIDADdeBX=Derecho]	-,160	,253	-,361	-,129	,609	-,245		
	[LATERALIDADdeBX=Izquierdo]	-,743	,314	-,159	-,039	-,106	,203		
	[cT=T1c]	,108	,376	-,253	-,112	-,514	,494		
	[cT=T2a]	,125	-,052	-,149	-,014	,136	-,349		
	[cT=T2b]	-,093	,182	,352	-,382	-,065	,343		
	[cT=T2c]	-,757	,508	-,175	,271	-,255	,150		
	[cN=N0]	,347	,364	,344	-,198	,232	-,318		
	[cN=N1]	-,358	-,107	,572	,061	,376	-,401		
	[cN=Nx]	,360	,393	-,113	-,274	-,342	,072		
	[miT=miT 2]	-,119	,522	,180	,105	-,162	,189		
	[miT=miT 3]	,129	-,240	-,241	,002	-,072	-,230		
	[miT=miT3a]	-,280	,407	-,262	-,563	,382	-,418		
	[miT=miT3b]	-,073	,209	,418	,396	,283	,338		
	[miN=miN 0]	-,279	,447	-,306	,198	,306	-,257		
	[miN=miN 1]	-,174	-,348	,283	,520	,526	-,140		
	EDAD	-,669	-,283	,103	-,289	-,119	-,224		
	DENSIDADPSA	-1,034	-,059	,276	,117	,468	-,468		
	VOLUMENPROSTATAc.c	-,273	,006	,380	-,409	-,254	,482		
	BRIGANTI	-,389	-,218	,292	-,037	-,250	,193		
Capa oculta 1	(Sesgo)							,459	-,757
	H(1:1)							-1,967	,578
	H(1:2)							,364	-,620
	H(1:3)							,845	-,322
	H(1:4)							-,731	,065
	H(1:5)							,550	-1,005
	H(1:6)							-,793	,141

Estimaciones de parámetro

Predictor		Pronosticado<br>Capa de salida<br>[GRUPODERIESGO=Intermedio]
Capa de entrada	(Sesgo)	
	[PSAALDX=2]	
	[PSAALDX=4]	
	[PSAALDX=5]	
	[PSAALDX=6]	
	[PSAALDX=7]	
	[PSAALDX=8]	
	[PSAALDX=9]	
	[PSAALDX=10]	
	[PSAALDX=11]	
	[PSAALDX=12]	
	[PSAALDX=13]	
	[PSAALDX=16]	
	[PSAALDX=19]	
	[PSAALDX=20]	
	[PSAALDX=21]	
	[PSAALDX=22]	
	[PSAALDX=36]	
	[ISUPBX=1]	
	[ISUPBX=2]	
	[ISUPBX=3]	
	[ISUPBX=4]	
	[ISUPBX=5]	

	[LATERALIDADdeBX=Bilateral]	
	[LATERALIDADdeBX=Derecho]	
	[LATERALIDADdeBX=Izquierdo]	
	[cT=T1c]	
	[cT=T2a]	
	[cT=T2b]	
	[cT=T2c]	
	[cN=N0]	
	[cN=N1]	
	[cN=Nx]	
	[miT=miT 2]	
	[miT=miT 3]	
	[miT=miT3a]	
	[miT=miT3b]	
	[miN=miN 0]	
	[miN=miN 1]	
	EDAD	
	DENSIDADPSA	
	VOLUMENPROSTATAc.c	
	BRIGANTI	
Capa oculta 1	(Sesgo)	,015
	H(1:1)	1,357
	H(1:2)	-,145
	H(1:3)	-,346
	H(1:4)	,649
	H(1:5)	,579
	H(1:6)	,154

Clasificación

Ejemplo	Observado	Pronosticado			
		Alto	Bajo	Intermedio	Porcentaje correcto
Entrenamiento	Alto	17	0	0	100,0%
	Bajo	0	0	2	0,0%
	Intermedio	0	0	10	100,0%
	Porcentaje global	58,6%	0,0%	41,4%	93,1%
Pruebas	Alto	6	0	1	85,7%
	Bajo	0	0	0	0,0%
	Intermedio	1	0	7	87,5%
	Porcentaje global	46,7%	0,0%	53,3%	86,7%

Variable dependiente: GRUPO DE RIESGO (D´amico)

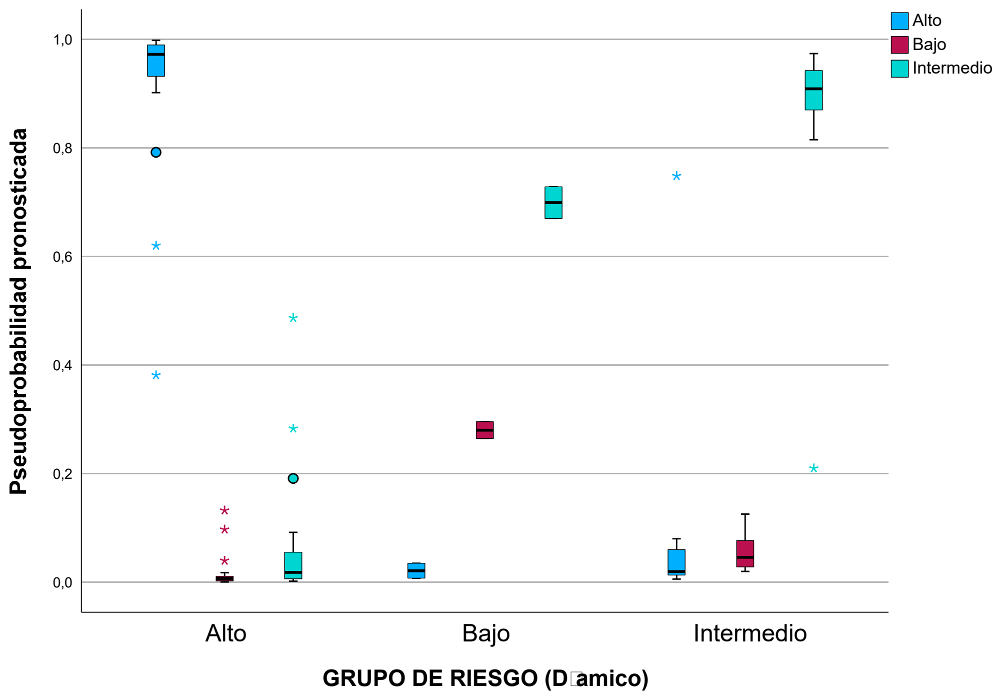

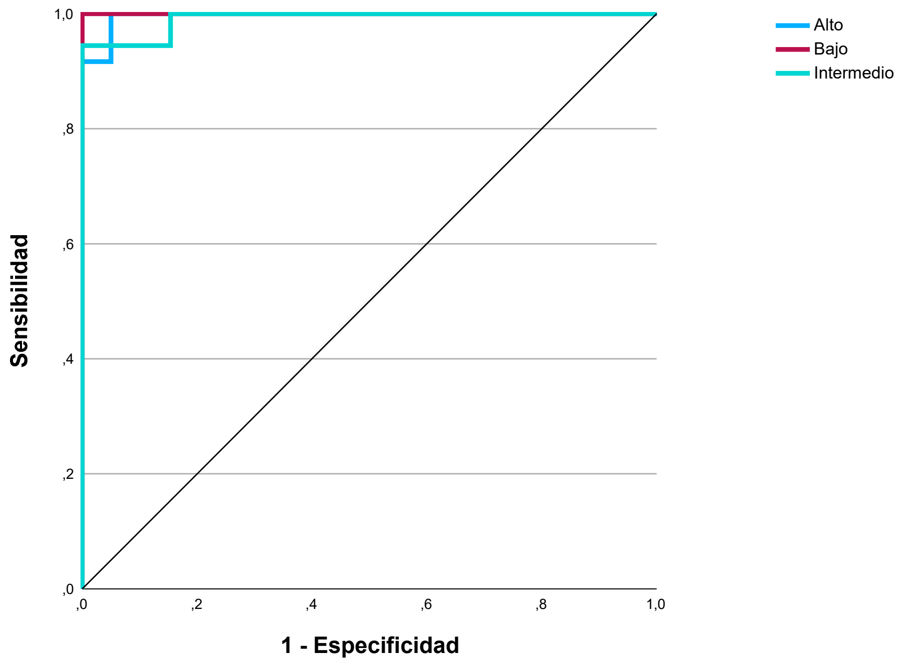

Variable dependiente: GRUPO DE RIESGO (D´amico)

Área bajo la curva

		Áreas
GRUPO DE RIESGO (D´amico)	Alto	,996
	Bajo	1,000
	Intermedio	,991

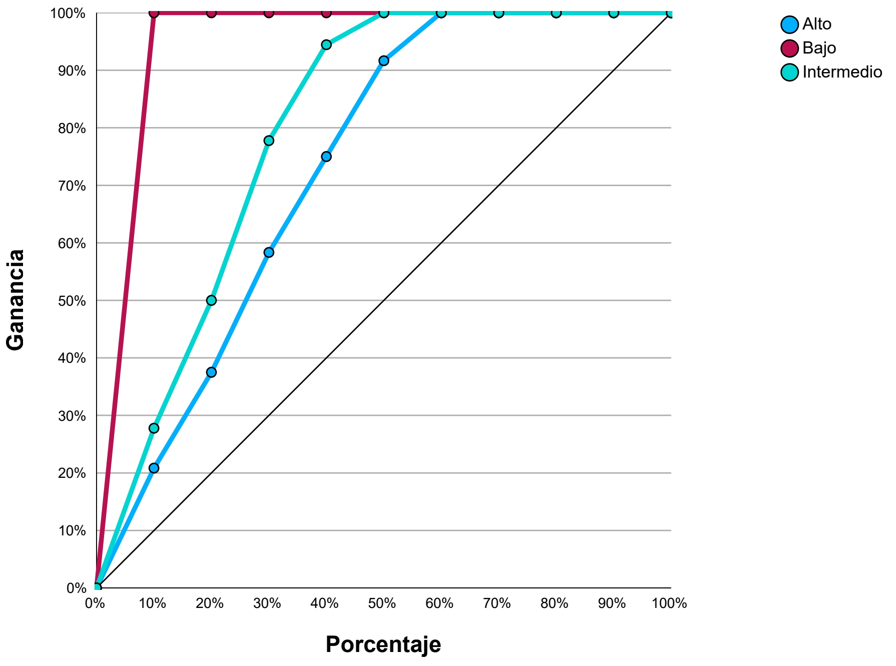

Variable dependiente: GRUPO DE RIESGO (D´amico)

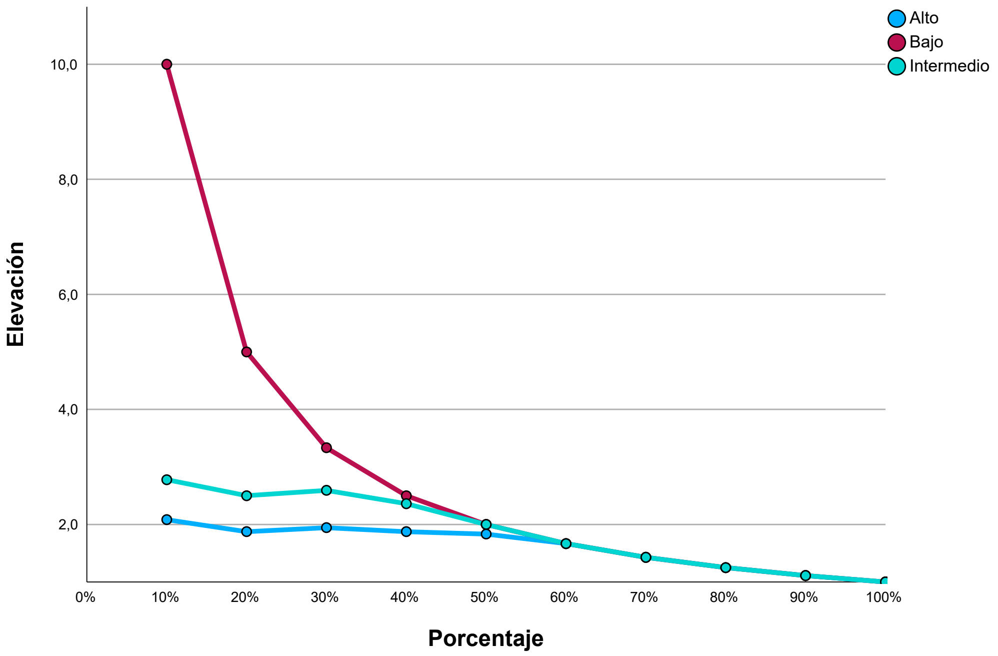

Variable dependiente: GRUPO DE RIESGO (D´amico)

Importancia de las variables independientes

	Importancia	Importancia normalizada
PSA AL DX	,092	45,4%
ISUP BX	,174	85,7%
LATERALIDAD de BX	,042	20,7%
Estadio clínico segun TNM	,052	25,5%
Estadio clínico ganglionar	,058	28,7%
miT	,047	23,0%
miN	,011	5,6%
EDAD	,154	76,0%
DENSIDAD PSA	,202	100,0%
VOLUMEN PROSTATA c.c.	,092	45,6%
BRIGANTI	,076	37,7%

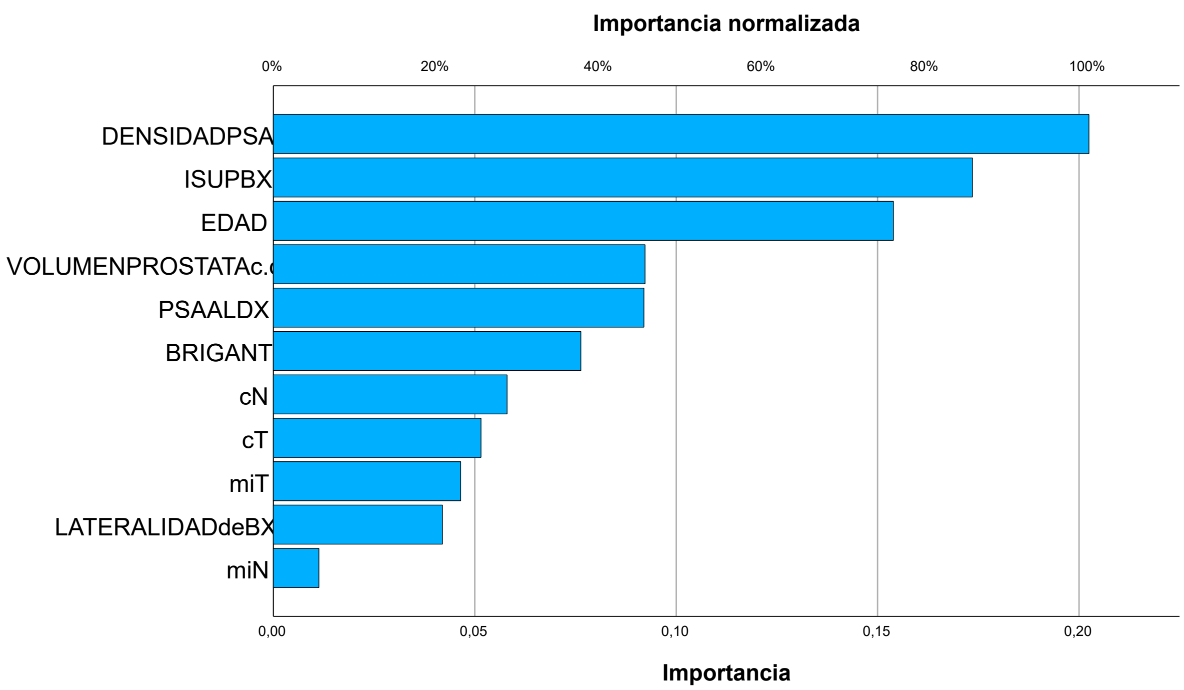

Importancia normalizada
0%
20%
40%
60%
80%
100%
DENSIDADPSA
ISUPBX
EDAD
VOLUMENPROSTATAc.
PSAALDX
BRIGANT
cN
cT
miT
LATERALIDADdeBX
miN
0,00
0,05
0,10
0,15
0,20
Importancia
