# Supplementary material for: Prioritising Data Quality Governance for AI in Prostate Cancer: A Methodological Proof-of-Concept Study Using Neural Networks for Risk Stratification
Source: Diagnostics (Basel). 2026 May 10;16(10):1454. doi: 10.3390/diagnostics16101454 (PMC13205476; doi:10.3390/diagnostics16101454)
Supplement: Supplementary file 1 [file diagnostics-16-01454-s001.zip › s6 OUTPUT20_80.pdf]

## Perceptrón multicapa

### Advertencias

| |
|---|
| Uno o más casos de la muestra de la prueba o la muestra reservada tienen un facto o valores de variable dependientes que no se producen en la muestra de entrenamiento. Estos casos se excluyen del análisis. |

### Resumen de procesamiento de casos

| | | N | Porcentaje |
|---|---|---|---|
| Ejemplo | Entrenamiento | 34 | 79,1% |
| | Pruebas | 9 | 20,9% |
| Válido | | 43 | 100,0% |
| Excluido | | 6 | |
| Total | | 49 | |

### Información de red

| | | | |
|---|---|---|---|
| Capa de entrada | Factores | 1 | PSA AL DX |
| | | 2 | ISUP BX |
| | | 3 | LATERALIDAD de BX |
| | | 4 | Estadio clínico segun TNM |
| | | 5 | Estadio clínico ganglionar |
| | | 6 | miT |
| | | 7 | miN |
| | Covariables | 1 | EDAD |
| | | 2 | DENSIDAD PSA |
| | | 3 | VOLUMEN PROSTATA |

| | | | |
|---|---|---|---|
| | | 4 | c.c. BRIGANTI |
| | Número de unidades[a] | | 43 |
| Capas ocultas | Método de cambio de escala para las covariables | | Estandarizados |
| | Número de capas ocultas | | 1 |
| | Número de unidades en la capa oculta 1[a] | | 9 |
| | Función de activación | | Tangente hiperbólica |
| Capa de salida | Variables dependientes | 1 | GRUPO DE RIESGO (D´amico) |
| | Número de unidades | | 2 |
| | Función de activación | | Softmax |
| | Función de error | | Entropía cruzada |

a. Se excluye la unidad de sesgo

## Resumen del modelo

| | | |
|---|---|---|
| Entrenamiento | Error de entropía cruzada | ,161 |
| | Porcentaje de pronósticos incorrectos | 0,0% |
| | Regla de parada utilizada | 1 paso(s) consecutivo(s) sin disminución del error[a] |
| | Tiempo de entrenamiento | 0:00:00,01 |
| Pruebas | Error de entropía cruzada | ,001 |
| | Porcentaje de pronósticos incorrectos | 0,0% |

Variable dependiente: GRUPO DE RIESGO (D´amico)

a. Los cálculos de error se basan en la muestra de comprobación.

**Estimaciones de parámetro**

| Predictor | | Pronosticado | | | | | | | | |
|---|---|---|---|---|---|---|---|---|---|---|
| | | Capa oculta 1 | | | | | | | | |
| | | H(1:1) | H(1:2) | H(1:3) | H(1:4) | H(1:5) | H(1:6) | H(1:7) | H(1:8) | H(1:9) |
| Capa de entrada | (Sesgo) | -,508 | ,066 | ,042 | ,497 | ,312 | ,550 | ,515 | ,111 | -,379 |
| | [PSAALDX=2] | -,112 | -,221 | -,206 | -,099 | -,234 | -,159 | -,392 | -,079 | ,146 |
| | [PSAALDX=4] | ,223 | -,060 | -,015 | ,502 | ,140 | -,299 | ,440 | -,276 | -,246 |
| | [PSAALDX=5] | -,387 | ,313 | -,233 | -,069 | -,319 | ,301 | ,362 | ,098 | ,268 |
| | [PSAALDX=6] | -,357 | -,244 | ,261 | ,707 | ,218 | ,160 | ,508 | -,564 | -,218 |
| | [PSAALDX=7] | ,343 | -,305 | -,175 | -,269 | ,149 | ,335 | -,033 | ,310 | -,362 |
| | [PSAALDX=8] | -,180 | ,303 | -,534 | -,186 | ,025 | ,221 | ,492 | ,000 | ,183 |
| | [PSAALDX=10] | -,055 | ,228 | ,533 | -,490 | -,346 | ,278 | -,539 | ,187 | -,026 |
| | [PSAALDX=11] | -,484 | -,042 | -,132 | ,510 | -,264 | -,360 | ,346 | ,252 | -,017 |
| | [PSAALDX=12] | ,929 | -,070 | ,301 | -1,123 | -,385 | -,608 | ,098 | ,412 | -,535 |
| | [PSAALDX=13] | -,261 | -,440 | -,105 | -,333 | ,328 | 5,893E-5 | ,434 | -,198 | ,355 |
| | [PSAALDX=16] | ,526 | -,103 | 1,031 | -,198 | -,016 | -,157 | -,238 | ,921 | -,105 |
| | [PSAALDX=19] | ,867 | ,336 | ,761 | -,941 | -,323 | -,692 | ,357 | ,291 | ,267 |
| | [PSAALDX=20] | -,353 | ,428 | -,588 | ,359 | ,066 | ,136 | ,625 | -,213 | -,026 |
| | [PSAALDX=21] | -,281 | ,250 | ,257 | ,227 | ,325 | ,090 | ,124 | -,196 | ,367 |
| | [PSAALDX=36] | -,194 | ,118 | -,734 | ,812 | ,035 | -,145 | ,375 | -,407 | ,542 |
| | [PSAALDX=41] | ,079 | ,016 | -,076 | -,454 | -,375 | ,044 | -,121 | ,048 | ,457 |
| | [PSAALDX=48] | ,348 | -,175 | ,190 | ,222 | -,125 | -,459 | ,251 | ,460 | ,410 |
| | [PSAALDX=69] | ,436 | ,070 | -,301 | -,163 | -,395 | ,056 | ,089 | -,182 | ,207 |
| | [ISUPBX=1] | 1,034 | -,182 | ,874 | -1,147 | ,268 | -,349 | ,083 | ,692 | -,651 |
| | [ISUPBX=2] | ,288 | -,370 | ,518 | -,132 | -,590 | ,427 | ,293 | ,478 | -,572 |
| | [ISUPBX=3] | 1,363 | -,428 | 1,273 | -1,620 | -,514 | -,500 | ,076 | ,494 | ,249 |
| | [ISUPBX=4] | -1,576 | -,082 | -1,135 | 1,474 | ,343 | 1,288 | ,918 | -,972 | ,740 |
| | [ISUPBX=5] | -1,056 | -,336 | -,235 | ,583 | ,234 | ,327 | ,173 | -,164 | ,368 |
| | [LATERALIDADdeBX=Bilateral] | ,715 | -,381 | ,186 | -,507 | -,118 | -,679 | ,171 | ,755 | ,188 |
| | [LATERALIDADdeBX=Derecho ] | -,340 | -,184 | -,095 | ,825 | ,238 | ,272 | ,396 | -,418 | ,421 |
| | [LATERALIDADdeBX=Izquierdo] | -,758 | -,029 | -,465 | ,398 | ,042 | -,047 | -,085 | -,095 | ,010 |
| | [cT=T1c] | -,012 | ,411 | ,956 | -,377 | ,495 | ,423 | ,338 | ,099 | ,063 |
| | [cT=T2a] | ,037 | ,363 | ,690 | -,310 | ,050 | -,554 | ,147 | ,310 | -,399 |
| | [cT=T2b] | -,027 | -,189 | -,016 | -,278 | ,181 | -,058 | -,103 | -,226 | ,284 |
| | [cT=T2c] | ,009 | -,440 | -,399 | ,186 | ,095 | ,029 | -,245 | ,129 | ,119 |
| | [cT=T3a] | ,249 | -,404 | -,427 | ,086 | ,217 | -,023 | -,104 | ,030 | ,285 |
| | [cN=N0] | ,036 | ,301 | ,063 | -,022 | ,538 | ,244 | -,102 | -,364 | -,360 |
| | [cN=N1] | -,368 | -,031 | ,204 | ,351 | -,108 | ,187 | -,314 | -,642 | -,205 |
| | [cN=Nx] | ,001 | ,391 | ,608 | -,099 | -,237 | ,161 | -,490 | ,227 | ,364 |
| | [miT=miT 2] | ,131 | ,222 | ,597 | -,406 | ,317 | -,205 | ,310 | ,091 | ,082 |
| | [miT=miT3a] | -,399 | -,271 | ,561 | -,162 | -,461 | ,090 | -,311 | ,286 | ,385 |
| | [miT=miT3b] | -,161 | ,122 | -,704 | 1,439 | ,456 | ,413 | ,263 | -,959 | ,143 |
| | [miN=miN 0] | -,093 | ,268 | -,549 | ,611 | ,293 | ,119 | -,363 | ,128 | -,145 |
| | [miN=miN 1] | -,075 | ,156 | ,144 | -,287 | -,198 | ,339 | -,496 | ,095 | -,455 |
| | EDAD | -,427 | ,169 | -,605 | -,679 | ,409 | -,200 | ,303 | -,748 | ,487 |
| | DENSIDADPSA | -,966 | -,473 | -,361 | ,649 | ,409 | ,000 | ,417 | -,878 | -,488 |
| | VOLUMENPROSTATAc.c | -,177 | ,137 | -,177 | ,823 | -,240 | ,388 | -,080 | ,821 | ,454 |
| | BRIGANTI | -,622 | -,514 | -,216 | 1,096 | ,593 | ,345 | -,223 | -1,596 | ,427 |
| Capa oculta 1 | (Sesgo) | | | | | | | | | |
| | H(1:1) | | | | | | | | | |
| | H(1:2) | | | | | | | | | |
| | H(1:3) | | | | | | | | | |
| | H(1:4) | | | | | | | | | |
| | H(1:5) | | | | | | | | | |
| | H(1:6) | | | | | | | | | |
| | H(1:7) | | | | | | | | | |
| | H(1:8) | | | | | | | | | |
| | H(1:9) | | | | | | | | | |

## Estimaciones de parámetro

| Predictor | | Pronosticado<br>Capa de salida<br>[GRUPODERIESGO=Alto] | <br><br>[GRUPODERIESGO=Intermedio] |
|---|---|---|---|
| Capa de entrada | (Sesgo) | | |
| | [PSAALDX=2] | | |
| | [PSAALDX=4] | | |
| | [PSAALDX=5] | | |
| | [PSAALDX=6] | | |
| | [PSAALDX=7] | | |
| | [PSAALDX=8] | | |
| | [PSAALDX=10] | | |
| | [PSAALDX=11] | | |
| | [PSAALDX=12] | | |
| | [PSAALDX=13] | | |
| | [PSAALDX=16] | | |
| | [PSAALDX=19] | | |
| | [PSAALDX=20] | | |
| | [PSAALDX=21] | | |
| | [PSAALDX=36] | | |
| | [PSAALDX=41] | | |
| | [PSAALDX=48] | | |
| | [PSAALDX=69] | | |
| | [ISUPBX=1] | | |
| | [ISUPBX=2] | | |
| | [ISUPBX=3] | | |
| | [ISUPBX=4] | | |

| | | | |
|---|---|---|---|
| | [ISUPBX=5] | | |
| | [LATERALIDADdeBX=Bilateral] | | |
| | [LATERALIDADdeBX=Derecho ] | | |
| | [LATERALIDADdeBX=Izquierdo] | | |
| | [cT=T1c] | | |
| | [cT=T2a] | | |
| | [cT=T2b] | | |
| | [cT=T2c] | | |
| | [cT=T3a] | | |
| | [cN=N0] | | |
| | [cN=N1] | | |
| | [cN=Nx] | | |
| | [miT=miT 2] | | |
| | [miT=miT3a] | | |
| | [miT=miT3b] | | |
| | [miN=miN 0] | | |
| | [miN=miN 1] | | |
| | EDAD | | |
| | DENSIDADPSA | | |
| | VOLUMENPROSTATAc.c | | |
| | BRIGANTI | | |
| Capa oculta 1 | (Sesgo) | ,463 | -,618 |
| | H(1:1) | -1,753 | 2,079 |
| | H(1:2) | -,241 | ,935 |
| | H(1:3) | -1,521 | 1,904 |
| | H(1:4) | 1,502 | -1,468 |
| | H(1:5) | 1,087 | -1,556 |
| | H(1:6) | 1,056 | -,713 |

| | | | |
|---|---|---|---|
| | H(1:7) | ,031 | ,261 |
| | H(1:8) | -,783 | ,768 |
| | H(1:9) | ,712 | -1,079 |

## Clasificación

| Ejemplo | Observado | Pronosticado Alto | Intermedio | Porcentaje correcto |
|---|---|---|---|---|
| Entrenamiento | Alto | 22 | 0 | 100,0% |
| | Intermedio | 0 | 12 | 100,0% |
| | Porcentaje global | 64,7% | 35,3% | 100,0% |
| Pruebas | Alto | 5 | 0 | 100,0% |
| | Intermedio | 0 | 4 | 100,0% |
| | Porcentaje global | 55,6% | 44,4% | 100,0% |

Variable dependiente: GRUPO DE RIESGO (D´amico)

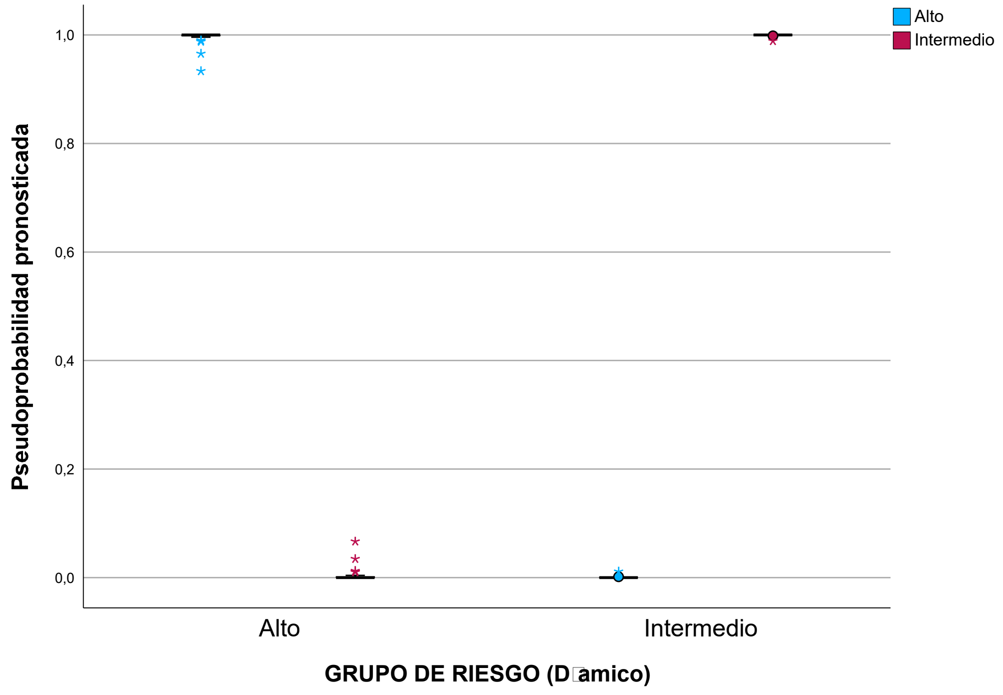

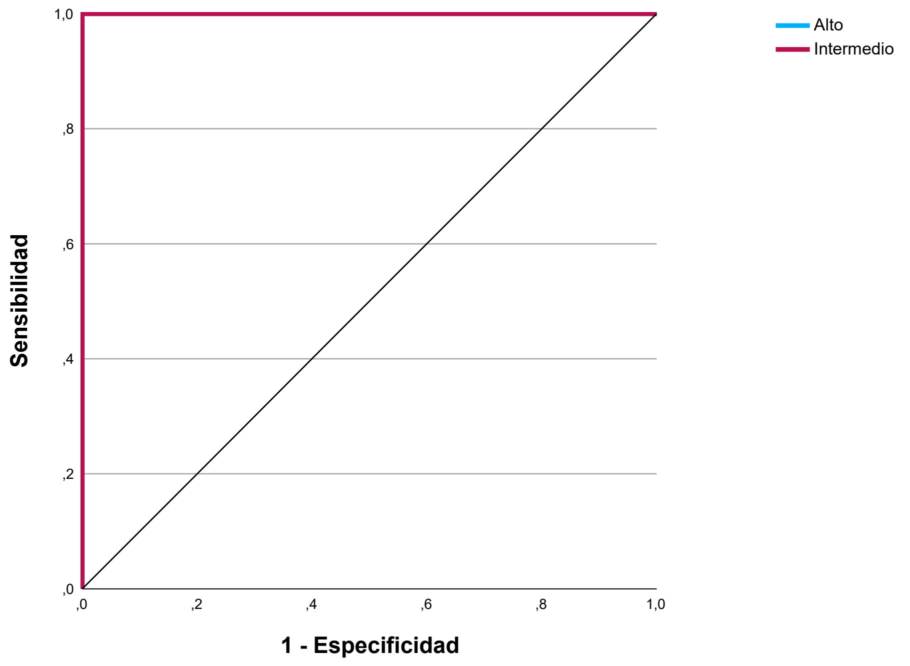


Variable dependiente: GRUPO DE RIESGO (D´amico)

# Área bajo la curva

| | | Áreas |
|---|---|---|
| GRUPO DE RIESGO (D´amico) | Alto | 1,000 |
| | Intermedio | 1,000 |

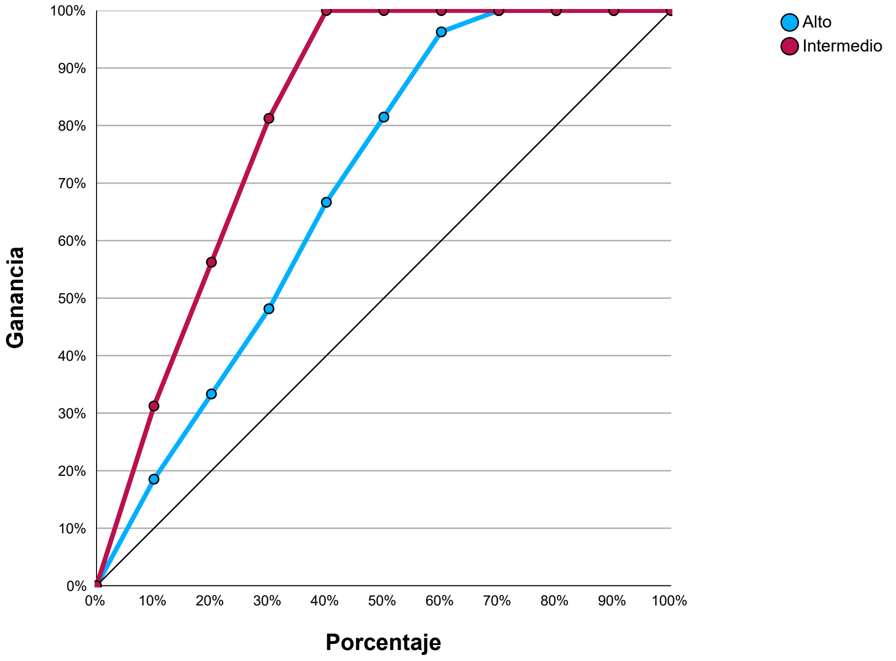


Variable dependiente: GRUPO DE RIESGO (D´amico)

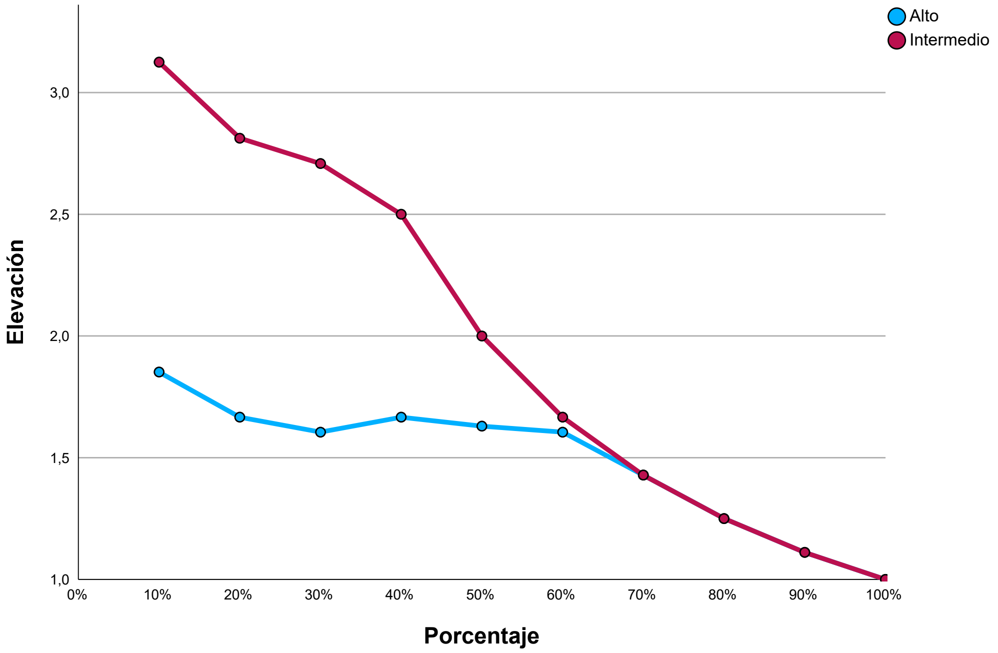


Variable dependiente: GRUPO DE RIESGO (D´amico)

## Importancia de las variables independientes

| | Importancia | Importancia normalizada |
|---|---|---|
| PSA AL DX | ,098 | 54,2% |
| ISUP BX | ,181 | 100,0% |
| LATERALIDAD de BX | ,067 | 37,0% |
| Estadio clínico segun TNM | ,053 | 29,1% |
| Estadio clínico ganglionar | ,025 | 14,1% |
| miT | ,071 | 39,1% |
| miN | ,032 | 17,9% |
| EDAD | ,098 | 54,0% |
| DENSIDAD PSA | ,127 | 70,2% |
| VOLUMEN PROSTATA c.c. | ,069 | 38,2% |
| BRIGANTI | ,180 | 99,3% |

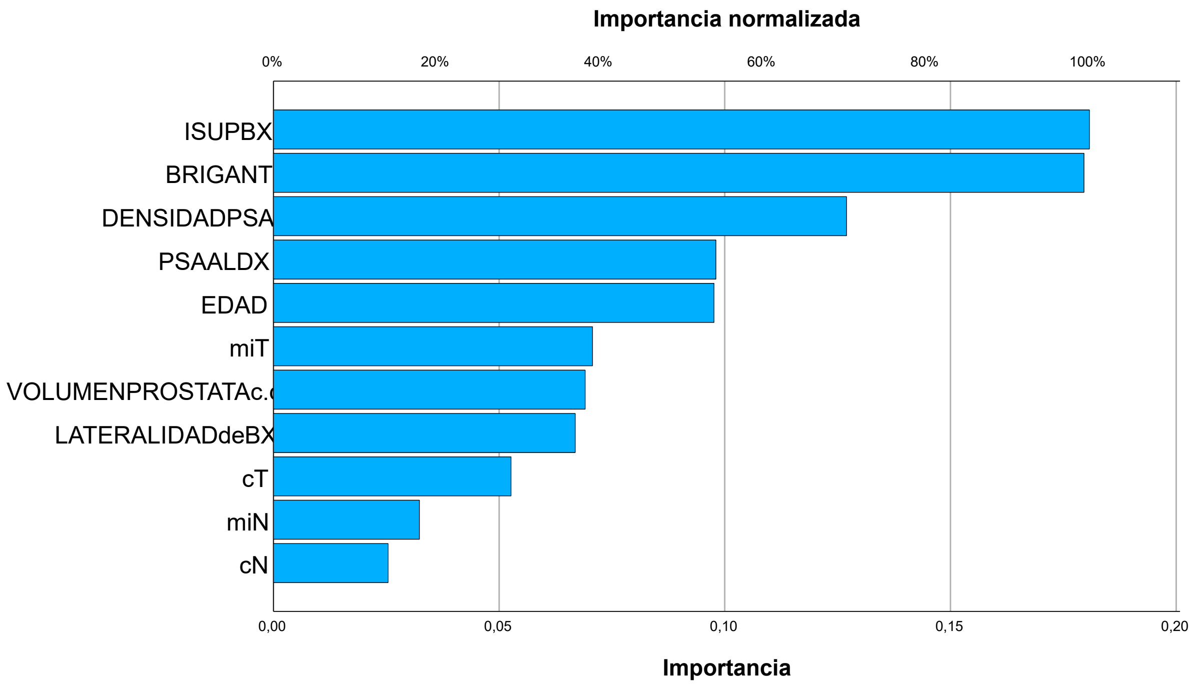
Importancia normalizada
0%
20%
40%
60%
80%
100%
ISUPBX
BRIGANT
DENSIDADPSA
PSAALDX
EDAD
miT
VOLUMENPROSTATAc.
LATERALIDADdeBX
cT
miN
cN
0,00
0,05
0,10
0,15
0,20
Importancia
