# Supplementary figures and images for: Prioritising Data Quality Governance for AI in Prostate Cancer: A Methodological Proof-of-Concept Study Using Neural Networks for Risk Stratification
Source: Diagnostics (Basel). 2026 May 10;16(10):1454. doi: 10.3390/diagnostics16101454 (PMC13205476; doi:10.3390/diagnostics16101454)

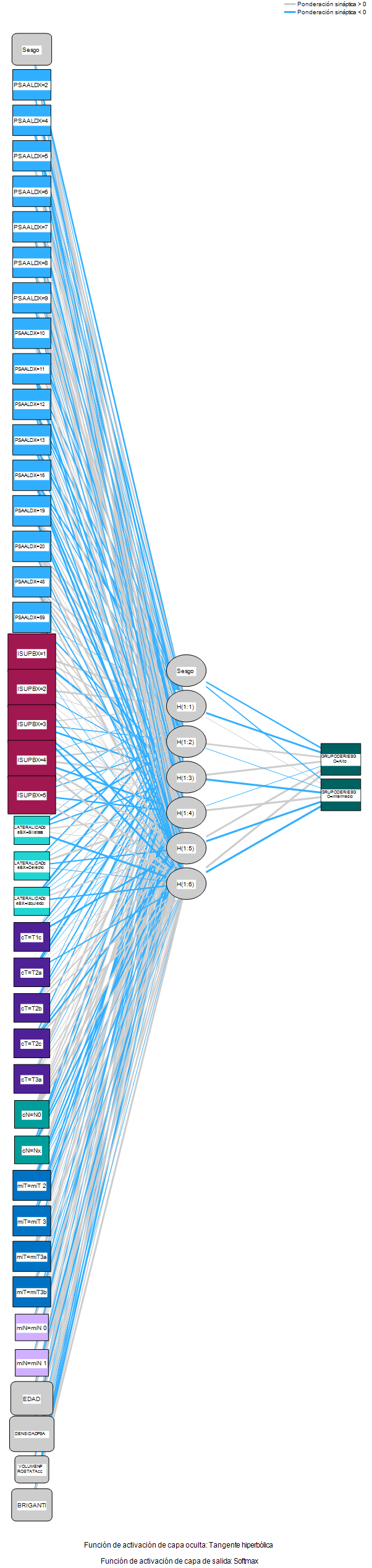

Supplement: Supplementary file 1 [file diagnostics-16-01454-s001.zip › S4 OUTPUT39_61.png]

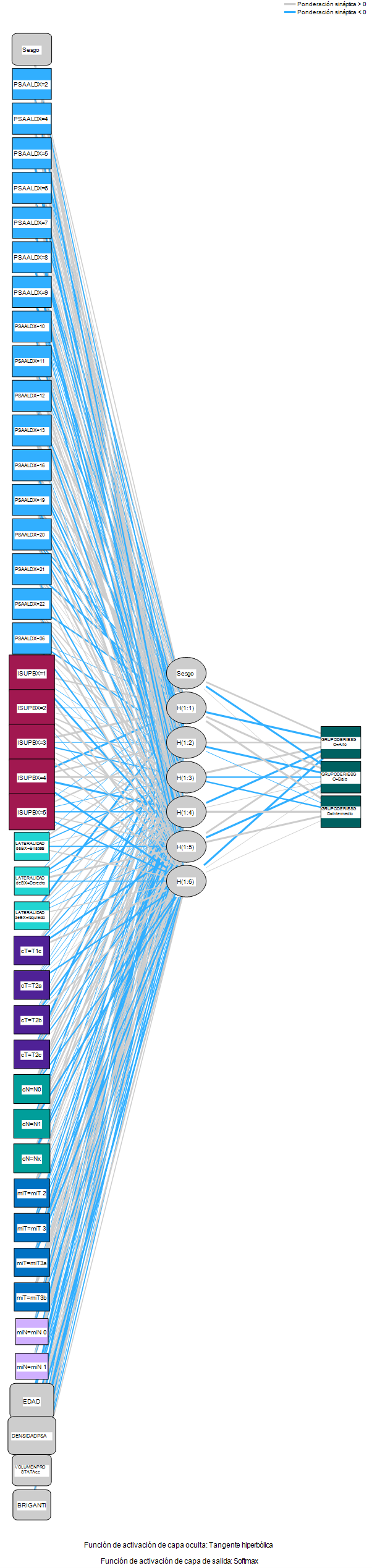

Supplement: Supplementary file 1 [file diagnostics-16-01454-s001.zip › S5 OUTPUT34_66.png]

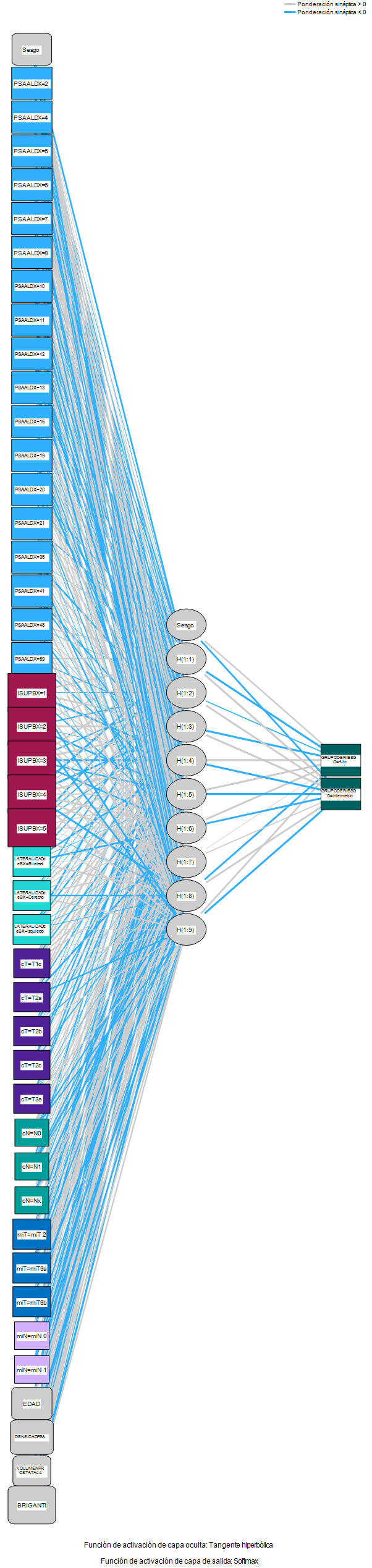

Supplement: Supplementary file 1 [file diagnostics-16-01454-s001.zip › s6 OUTPUT20_80.png]
